# A high-fat diet nutritional intervention reprograms cardiac metabolism and improves systolic function in a pig model of heart failure with reduced ejection fraction

**DOI:** 10.1007/s00395-026-01171-y

**Published:** 2026-03-27

**Authors:** Carlos Galán-Arriola, Daniel Pérez-Camargo, Alessia Ferrarini, Annalaura Mastrangelo, María Isabel Higuero-Verdejo, Gonzalo Javier López-Martín, Ana Devesa, Rocío Villena Gutiérrez, Miguel Fernández-Tocino, Anabel Díaz-Guerra, Lourdes Montero-Cruces, Manuel Carnero, Rodrigo Fernández-Jiménez, Valentin Fuster, Javier Sánchez-González, Borja Ibáñez

**Affiliations:** 1 Translational Laboratory for Cardiovascular Imaging and Therapy, Centro Nacional de Investigaciones Cardiovasculares Carlos III (CNIC), Madrid, Spain; 2 Centro de Investigación Biomédica en Red en Enfermedades Cardiovasculares (CIBERCV), Madrid, Spain; 3 San Carlos Clinic University Hospital, Madrid, Spain; 4 Mount Sinai Hospital, New York, NY USA; 5 Philips Healthcare, Madrid, Spain; 6 Cardiology Department, IIS-Fundación Jiménez Díaz Hospital, C/ Melchor Fernández Almagro, 3, 28029 Madrid, Spain

**Keywords:** Heart failure, High-fat diet, Mitochondria, Magnetic resonance imaging

## Abstract

Most forms of heart failure are characterized by a metabolic switch from the use of fatty acids to glucose as the main fuel source for ATP generation in the myocardium. Whether metabolic reprogramming is a therapeutic target remains controversial. In this study, heart failure with reduced ejection fraction (HFrEF) and metabolic switch (i.e., increased myocardial glucose uptake) was induced in pigs by generating viable dysfunctional myocardium secondary to progressive coronary artery stenosis. Pigs (*n* = 19) were then randomized to a high-fat diet (HFD, chow diet supplemented with 20% lard) or control diet (no supplementation) for two months. Pre- and post-nutritional treatment contrast-enhanced cardiac magnetic resonance (CMR) and ^18^FDG-PET/CT studies were performed. Hearts were then harvested for further analysis. LVEF significantly improved in pigs receiving the 2-month HFD (38% [33, 43] to 54% [47, 62], *p* = 0.036) but remained unchanged in control-diet pigs (36% [35, 45] to 41% [38, 43], *p* = 0.24). HFD-fed pigs had a smaller extent of fibrosis after the dietary intervention (late gadolinium enhancement 0.45% LV [0.17, 1.67] vs 6.23 [5.54, 9.57], *p* = 0.0047). On ^18^FDG-PET, a reversion of the metabolic reprogramming in the LAD-dysfunctional myocardium was observed only in HFD-fed pigs (0.46 counts [0.21, 0.65] vs 1.80 [1.53, 2.83], *p* = 0.016). Transmission electron microscopy of explanted hearts revealed less fragmented mitochondrial and a lower lipid droplet density in cardiomyocytes from HFD-fed pigs (38 per 10 µm3 [34, 50] vs 96 [78, 124], *p* = 0.022), and this was accompanied by increased expression of genes involved in fatty acid metabolism and downregulation of genes encoding glucose import proteins. In conclusion, in a large animal model of HFrEF secondary to myocardial dysfunction with a metabolic switch, a nutritional intervention based on HFD feeding was associated with a cardiac metabolic restoration of fatty acid substrate use, restoration of cardiomyocyte lipid trafficking and significantly improved systolic function.

## Introduction

Despite advances in treatment, heart failure (HF) remains a major contributor to global morbidity and mortality, and there is a need to identify therapies targeting nonredundant disease pathways [33].

The myocardium has the highest specific energy requirement of any tissue, phosphorylating and dephosphorylating the equivalent of 5 kg of ATP per day under steady-state conditions [2, 10]. The myocardium is able to meet this enormous energy demand by being a metabolic omnivore, able to use different fuels as the substrate for energy production. Under steady-state conditions, the primary energy source is β-oxidation of fatty acids since this yields significantly more ATP per molecule oxidized compared to oxidation of carbohydrates (glucose) or amino acids [10]. Most forms of heart failure (HF) are characterized by a myocardial metabolic switch from this reliance on fatty acids to the predominant use of glucose as the substrate for ATP generation [50]. This metabolic switch has historically been considered as a response to limited energy availability.

Emerging evidence suggests that diets low in carbohydrates, and thus with a higher percentage of calories derived from fat (or ketones if the levels of carbohydrates are extremely low), can be beneficial in HF caused by pressure overload (including systemic hypertension) by partially reversing the metabolic switch [49]. In a mouse model of cardiomyocyte-specific genetic ablation of the protease YME1L, resulting in fragmented mitochondria, we recently demonstrated that a high-fat diet (HFD) prevented the onset of dilated cardiomyopathy [55]. Taken together, this evidence suggests that the metabolic switch in HF could represent a novel therapeutic target. However, these small animal studies tested the preventive effect of fat-rich diets, and it is unknown if a nutritional intervention can restore cardiac function after manifest HF. There is also a lack of robust evidence from models with a physiology more similar to the human.

We recently developed a pig model of HF with reduced ejection fraction (HFrEF) secondary to a progressive LAD occlusion with a minimum LGE extension [32]. In this model, systolic function is progressively depressed while maintaining a viable myocardium (absence of transmural necrosis and presence of contractile reserve upon dobutamine challenge). An overt metabolic switch in these pigs is evidenced by ^18^fluorodeoxyglucose-position emission tomography (^18^FDG-PET) [32]. Here, we used this highly translatable model to test whether a nutritional intervention based on a significant increase in the proportion of calories derived from fat is able to reverse the HF metabolic switch and improve cardiac function evaluated with state-of-the-art imaging modalities.

## Methods

### Animals and study design

**Fig. 1:**
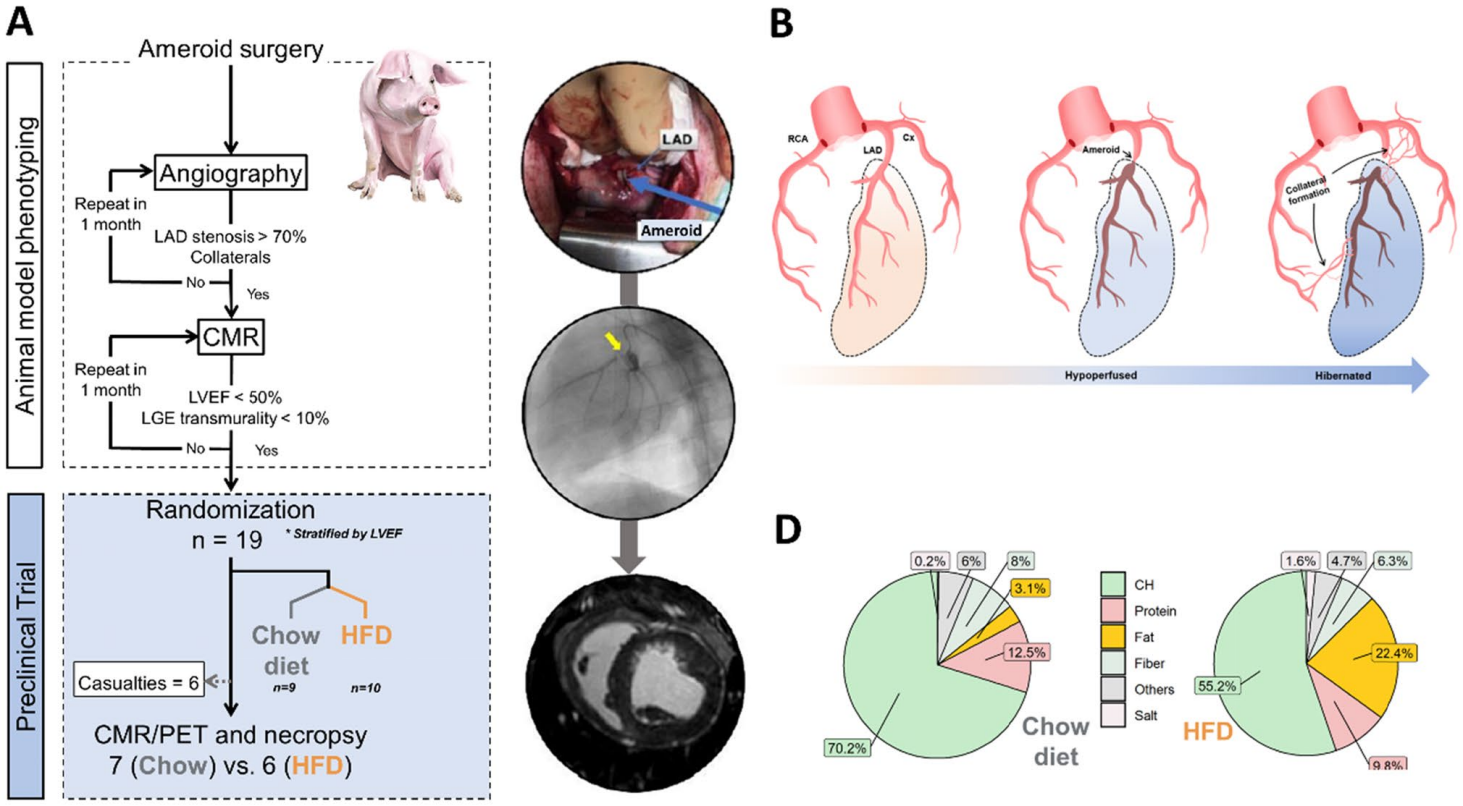
Study design and model phenotyping. **A** Study design and sample size, showing the phenotyping algorithm and indicating casualties and excluded animals. Representative pictures show ameroid surgery, angiography with LAD flux maintained by collaterals from the Cx coronary artery, and a CMR LGE sequence showing endomyocardial fibrosis. **B** Schematic representation of viable dysfunctional myocardium development in the ameroid ring model. **D** Chow diet and high-fat diet composition. CMR: cardiac magnetic resonance; HFD: high-fat diet; LAD: left anterior descending; LVEF: left ventricular ejection fraction; PET: positron emission tomography

All the animals used and experiments performed followed the Directive 2010/63/EU with local protocol number. PROEX103/19 The study design, including the phenotyping and preclinical trial phases, is summarized in Fig. 1A. Experiments were conducted on 2-month-old castrated male Large White pigs. Using a previously published surgical procedure [32], an ameroid ring was placed around the proximal left anterior descending (LAD) coronary artery. Two months after surgery, animals were phenotyped by multimodality imaging: invasive coronary angiography, gadolinium-enhanced cardiac magnetic resonance (CMR), and ^18^FDG-PET to check for the presence of metabolic reprogramming myocardium. Animals fulfilling the predefined potentially LV viable phenotype criteria (severe stenosis (≥ 70%) of the LAD plus coronary collaterals, and systolic dysfunction (left ventricular ejection fraction (LVEF) < 50%) without transmural late gadolinium enhancement (LGE), and avid glucose uptake in the myocardium at risk, Fig. 1B) were randomized 1:1 to 2 months on a regular chow diet or a high-fat diet (HFD); researchers were blinded to the dietary allocation until the end of the analyses (only animal facility staff knew). In the regular chow diet, fat contributed 3.1% of the calorie content; in contrast, the HFD consisted of 80% regular chow plus 20% lard, resulting in 22.4% of calories coming from fat. The two diets were isocaloric (Fig. 1D). After the 2-month dietary intervention, animals underwent a final imaging session consisting of a multiparametric CMR exam and a cardiac ^18^FDG-PET study. The pigs were then euthanized using i.v. sodium pentobarbital in overdose (10 mL, Dolethal®), and hearts were excised for additional ex vivo analyses.

### Ameroid ring implant surgery

The surgical procedure was as reported previously [32]. Briefly, animals were anesthetized by i.m. injection of ketamine (20 mg/kg), xylazine (2 mg/kg), and midazolam (0.5 mg/kg), and anesthesia was maintained with inhaled sevoflurane (3–4%). Analgesia was maintained during surgery by continuous i.v. infusion of fentanyl solution. A minimal left thoracotomy was performed between the third and fourth intercostal space. The pericardium was opened, and the LAD artery was located and exposed by producing a traction point on the left atrial appendage. The LAD was then carefully dissected free, and the ameroid ring was placed around the proximal segment. Subsequently, the left atrial traction was removed, hemostasis was achieved, the thoracotomy was closed flat, and the animal was allowed to recover. After the procedure, pigs received dual antiplatelet therapy (aspirin 100 mg plus clopidogrel 75 mg, daily) until the end of the study to reduce the incidence of coronary thrombosis, as well as oral metoprolol (50 mg/d) for the first 45 days (or until the demonstration of phenotype first) to reduce the incidence of malignant arrhythmias.

### Coronary angiography

The methodology used was as described before [32]. Briefly, anesthesia was induced by intramuscular injection of ketamine (20 mg/kg), xylazine (2 mg/kg), and midazolam (0.5 mg/kg) and maintained by continuous intravenous infusion of ketamine (2 mg/kg/h), xylazine (0.2 mg/kg/h), and midazolam (0.2 mg/kg/h) and endotracheally intubated for mechanical ventilation. The femoral artery was accessed by the Seldinger technique, and a 6-French sheath was inserted. Pigs were anticoagulated with 150 IU/kg of i.v. heparin, and a 5-French coronary diagnostic catheter was inserted via the femoral sheath and docked at the origin of the left coronary artery. Left coronary angiography was recorded with iobitridol contrast (Xenetic, Guebert, Villepinte, France) to check for LAD stenosis and the development of homocoronary collaterals. Right coronary artery angiography was also recorded to assess the presence of heterocoronary collaterals. Once the exam was completed, the catheter material was removed, and the animal was allowed to recover.

### Cardiac magnetic resonance imaging

All studies were performed with a Philips 3-T Achieva Tx whole body scanner (Philips Healthcare, Best, the Netherlands) equipped with a 32-element phased-array cardiac coil and the animals sedated and maintained with the same protocol detailed for the catheterization. The CMR protocol included a standard segmented cine steady-state free-precession (SSFP) sequence to provide high-quality anatomical references and T1-weighted LGE sequences. The imaging parameters for the cine SSFP sequences were as follows: field of view (FOV), 280 × 280 mm; slice thickness, 6 mm with no gaps; repetition time (TR) 2.8 ms; echo time (TE), 1.4 ms; flip angle, 45°; cardiac phases = 30; voxel size, 1.8 × 1.8 mm; and number of excitations (NEX) = 3. LGE imaging was performed 10–15 min after intravenous administration of 0.2 mM/kg gadopentetate dimeglumine contrast agent using a 3D inversion recovery spoiled turbo field echo sequence (TR/TE/Flip angle = 2.4 ms/1.13 ms/10°) with an isotropic resolution of 1.5 × 1.5 × 1.5 mm^3^ on a FOV of 340 × 340 ×  mm^3^ in the FH, LR, and AP directions. Data were acquired in mid-diastole with a 151.2 ms acquisition window. Acquisition was accelerated using a net SENSE factor of 2.25 (1.5 × 1.5 in the AP and LR directions) with a bandwidth of 853 Hz per pixel. Inversion time was adjusted before acquisition using a look-locker scout sequence with different inversion times to ensure proper nulling of the healthy myocardium signal. For the analysis, 3D volume was reconstructed in short-axis view with a slice thickness of 6 mm. For LGE transmurality, the average of the LAD-irrigated AHA segments (1, 2, 7, 8, 13, and 14) was considered.

### Positron emission tomography/computed tomography (PET/CT)

All studies were performed with a GEMINI TF 64-slice PET/CT scanner (Philips Healthcare, Best, the Netherlands). In the 24 h before the scan, animals were fasted, with free access to drinking water. On the day of the scan, blood glucose was normalized to 100–150 mg/dL. To achieve this, blood drops were obtained and glucose measured with the CONTOURM PLUS Blood Glucose Monitoring System. If the glucose concentration was below 100 mg/dL, the animal was given an i.v. bolus injection of a 50% glucose solution (5 mL for each 10 mg/dL interval below 100 mg/dL). If the glucose concentration was above 150 mg/dL, the measurement was repeated at 10–15-min intervals until it had normalized. Animals then received an i.v. injection of FDG radiotracer (10 mCi per study), and were left for 40 min to allow biodistribution. After acquisition, a contrast-enhanced cardiac CT scan was performed for radioactive signal-anatomical superposition. PET data were analyzed with dedicated software (IntelliSpace Portal, Philips Healthcare, Best, The Netherlands) by segmenting the heart and placing a ROI on the irrigated LAD (distal to the ameroid ring) and on the remote areas; data were normalized to count values in the right ventricle.

### Plasma sampling and lipid profile

Prior to randomization and at the end of the study, blood samples were collected from the ear vein in heparin tubes and plasma was obtained after 30 min of centrifugation at 1500 rpm. After that, samples were analyzed by Dimension RxL Max machine (Siemens) for basic biochemistry and lipid profile. Cholesterol was measured by cholesterol oxidase, esterase, and peroxidase reaction. Triglycerides were assessed by enzymatic reaction as well, LDL and HDL by direct measure. ALT/GPT, AST/GOT with UV with P5P method. GGT by γ-glutamyl-carboxy-nitroanilide and bilirubin were measured by Jendrassik Grof method.

### Histopathology

At the end of the protocol, animals were sedated and euthanatized by i.v. pentobarbital in overdose. The heart was excised, and blood removed using abundant saline at room temperature. For histological studies, samples from the anterior wall (distal to the ameroid ring) and remote (posterior) areas were fixed in 4% formalin at least 48 h and then transferred to 70% ethanol. After paraffin embedding, 4-micron sections were cut and stained with hematoxylin and eosin (HE), Masson trichrome (MT), and Sirius Red (SR). SR-stained sections were scanned, and collagen was quantified in 20 × 20 magnified images using a modified macro [20].

### Transmission electron microscopy

Additional myocardial samples from anterior and remote areas were maintained in 4% glutaraldehyde in 10% PFA solution for 24–48 h. Tissues were then post-fixed in 1% osmium tetroxide in water for 1 h at room temperature. Samples were washed with water and then block stained with 0.5% uracil acetate in water for 10 min. Following this, samples were dehydrated through a graded series of aqueous ethanol solutions (30%, 50%, 70%, 75%, and 100%) and finally to acetone. The samples were then included in Durcupan epoxy resin through stepwise incubation in resin–acetone mixtures of increasing resin content (1:3, 3:1) and finally in pure resin. Resin-included samples were polymerized in an oven at 60 ºC for 48 h. Samples were ultra-thin sliced (60 nm) on a Leica Ultracut ultramicrotome S and deposited on 200 mesh copper grids. The grids were counterstained with uranyl acetate and lead citrate. Images were obtained with a Jeol Jem1010 (80 kV) transmission electron microscope linked to a Gatan camera (Orius 200 SC model) and processed with Digital Micrograph software. Individual images were acquired at 6000, 10,000, 20,000, and 40000X magnifications, and mitochondria size and shape were evaluated with ImageJ software 1.52p Wayne Rasband from USA National Institute of Health (NIH) as previously described [14].

### Western blot analysis of protein expression

Cardiac samples from the anterior myocardial region were lysed in RIPA buffer supplemented with a protease and phosphatase inhibitor cocktail. Protein content was quantified with the Bio-Rad BCA protein assay. Proteins were separated by SDS–polyacrylamide gel electrophoresis and transferred to nitrocellulose membranes. After blocking, the membranes were incubated and probed with antibodies to PGC1-alpha (Cell Signaling, 3738S), MFN2 (Abcam, 23,707), and Vinculin (Abcam, ab8245) at 4 °C overnight according to the manufacturer's instructions. After incubation with appropriate secondary antibodies and further washes, bound proteins were revealed by enhanced chemiluminescence. Quantitative densitometric analysis was performed using ImageJ Fiji software.

### PCR analysis of transcript expression

RNA extraction was performed using RNeasy plus mini kit. Tissue was digested using 300 μl of TRIzol reagent and homogenized using a TissueLyser 15 min 1/50 s frequency. Samples were incubated 5 min at RT. The sample volume did not surpass 10% of the TRIzol volume used. 0.15 ml of chloroform per 300 μl of TRIzol was added, and samples were energetically vortexed 15 s and incubated at RT 3 min. Centrifugation at 12000 x g at 4ºC 15 min was carried out. RNA remained in the upper aqueous phase after centrifugation, which was transferred into a fresh tube. Following kit protocol, RNA was eluted in 30 μl of mqH2O and quantified as described with DNA.

Isolated RNA was reverse-transcribed into cDNA using Applied Biosystems™ High-Capacity cDNA Reverse Transcription Kit using 600 ng of RNA. The cDNA was analyzed by qPCR using the SYBRTM green PCR master mix, using the following program: step 1 (50ºC, 2 min), step 2 (95ºC, 10 min), step 3 (95ºC, 15 s and 60ºC, 1 min; × 39 cycles), step 4 (65 to 95ºC with 0.5ºC increment each 5 s). Primers used for qPCR are available upon request.

Relative expression of target genes was calculated using the 2–∆∆Ct method and normalized using hypoxanthine phosphoribosyltransferase (HPRT) housekeeping gene.

### Metabolomic analysis by gas chromatography–mass spectrometry

Myocardial samples from LAD and remote areas were homogenized in ice-cold PBS on ice and an aliquot of 100 μL of homogenate was collected. Briefly, 10 mg of tissue sample was lysed in PBS in a 1:20 ratio (sample:solvent) with FastPrep-24 5G instrument (MP Biomedicals, USA), with CoolPrep adapter, for 60 s at 4 m/sec, with a 30 s resting pause after 30 s. The process was repeated twice. Homogenates were collected and metabolites were extracted by adding 250 μL of ACN (1:2.5 v/v) with 10 ppm of 4-nitrobenzoate. Samples were then vortexed for 2 min and incubated on ice for 20 min followed by centrifugation for 20 min at 4 °C, at 12,000 rpm. A 125 μL aliquot of the supernatant was collected, placed into GC–MS vial with 300 µL insert and dried out in a Speedvac. Dried supernatant was derivatized following oximation and trimethylsilylation. Briefly, 10 μL of *O*-methoxyamine HCl in pyridine was added to the dried extract, vortex-mixed for 2 min and incubated in darkness during 16 h at room temperature. Following this step, 10 μL of BSFTA + 1% TMCS was added. Samples were vortex-mixed for 5 min and incubated at 70 degrees by constant shaking (1-h, 400 rpm) and allowed to cool down for one hour at room temperature. Finally, 50 μL of heptane containing 10 ppm of methyl stearate was added to the vial and centrifuged for 10 min at 2000 rpm at 15 degrees prior to GC–MS analysis.

One microliter of the derivatized metabolites from tissue samples was analyzed using the Agilent 8890 GC system coupled to the LECO BT GC-TOF–MS (LECO Corporation) and injected onto a Restek Rxi-5 ms (30 m × 0.25 mm i.d. × 0.25 μm coating) in split mode (ration 5:1). The GC and MS parameters were set as follows: 99.9% helium at a flow rate of 1 ml/min; the oven temperature was set at 60 °C for 1 min, then ramped by 10 °C/min up to 300 °C and held constant for 4 min; inlet at 250 °C transfer line temperature at 280 °C; electron impact (EI) ionization at 70 V and ion source temperature at 300 °C; solvent delay of 5.5 min; full scan mode (50–600 m/z) at a rate of 12 scans per second; total chromatographic separation time of 35 min.

Quality control samples (QCs) were run at the beginning of the analysis, at the end, and every 6 samples for quality assessment. To ensure the reliability and traceability of the results, routine maintenance and calibration of the equipment were performed before the analysis.

GC–MS data processing, including alignment, deconvolution, and identification, was performed using ChromaTOF Sync software (Version 1.151 from LECO Corporation). Identification of analytes detected by GC-TOF–MS was achieved by comparing the experimental spectra with the ones compiled in the NIST 2020 (version 2.4). Data quality was ensured through principal component analysis (PCA) of the samples and QCs, as well as the evaluation of the internal standards profile.

### Fatty oxidation activity assay

Fatty acid oxidation (FAO) was assessed by an assay kit (AssayGenie®, ref:BR000001). Myocardial tissue from the LAD area for each individual was lysed in cell lysis solution buffer for 15 min. After that, the lysate was centrifuged cold at 14,000 rpm for 5 min, obtaining the supernatant for the BCA protein assay method to determine the lysate protein concentration. After that, we plated all samples in duplicate and added 20 ul of control or reaction solution. Afterward, the plate was incubated for 60–120 min at 37 ºC and read at OD_492_ in a plate reader for evaluating the enzymatic activity of oxidation. Finally, FAO activity was calculated using the formula: FAO activity = ΔO.D. × 3.24. Enzyme activity is presented as units/μg proteins.

### Glucose, insulin plasma assay, and HOMA-IR calculation

For blood glucose measurement, an adapted version of the hexokinase–glucose-6-phosphate method by Kunst was used on the Dimension RxL Max analyzer (Siemens® Healthineers). Insulin levels were measured using a porcine-specific ELISA kit (ab273208, Abcam) following the manufacturer’s instructions. Briefly, standards and samples were incubated in 8-well strips for 2.5 h at room temperature, followed by sequential incubations with biotinylated antibody (1 h), streptavidin solution (45 min), and TMB substrate (30 min in the dark). The reaction was stopped, and absorbance was read at 450 nm. A standard curve was generated to determine insulin concentrations. HOMA-IR was calculated as follows:

HOMA-IR = (Glucose (mg/dL) × Insulin (μU/mL)) / 405. 

### Statistical analysis

Continuous variables are presented as median [interquartile range]. All data were analyzed with RStudio (RStudio Team (2015): Integrated Development for RStudio, Inc., Boston, MA), and graphics were generated and statistics calculated with the tidyverse package.

The primary outcome measure of the study was the between-group difference in final LVEF. Given both the lack of previous large animal studies testing the effect of HFD on HFrEF secondary to myocardial dysfunction and the high drop-out rate before the trial phase, we estimated sample size based on expected final LVEF of at least 6–7 animals per group at the end of the protocol[47]. The non-parametric Wilcoxon rank-sum test was used unpaired for the two-group comparison (regular chow diet vs HFD) and paired for the two-timepoint comparison (baseline vs 2 months).

## Results

A total of 19 pigs with LV dysfunction but potentially viable phenotype fulfilled the predefined criteria for HFrEF and entered the trial phase. These pigs were 1:1 randomized, with stratification by baseline LVEF, to control regular chow diet (*n* = 10) or HFD (n = 9). During the trial phase, 6 animals (3 in each arm) died from sudden cardiac death. Therefore, a total of 13 pigs completed the study and had a primary outcome measure available (HFD = 6, Chow = 7) (Fig. 1).

### Impact of a 2-month high-fat diet on body weight, circulating lipid concentrations, and liver function

Animals were weighed immediately before group assignation, and the randomization procedure was balanced to ensure no between-group difference in baseline body weight (HFD = 66.3 [58.3–72] kg, Chow = 69 [58.5–73] kg). After the 2-month dietary intervention, body weight was not significantly higher in the HFD group (HFD = 89.8 [83.3–111] kg, Chow = 94.5 [80–104] kg).

**Fig. 2:**
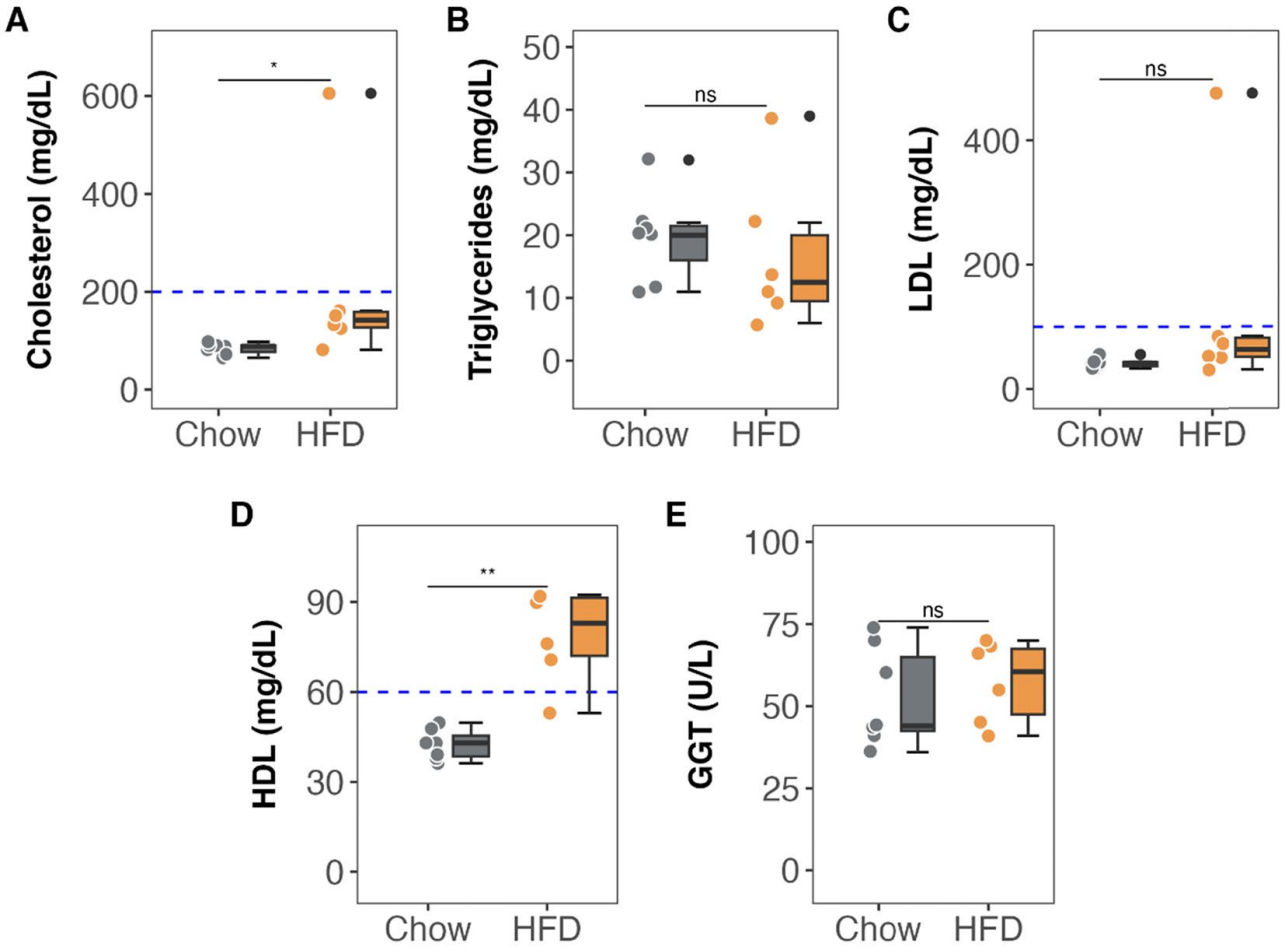
Lipid/hepatic profile after 2 months of high-fat diet administration. **A–D** Circulating plasma lipid parameters. **E** Circulating plasma liver GGT enzyme. Colored dots and lines indicate individual values for each group (gray for Chow diet and orange for HF diet). Tukey’s boxplots show median values (bold lines), interquantile range (boxes), and the remaining data distribution (whiskers). Asterisks indicate statistical significance as follows: ns: no significance, *p < 0.05, **p < 0.01, ***p < 0.001 (non-parametric Wilcoxon rank test). Sample size: *n* = 7 in Chow and n = 6 in HFD group. HFD, high-fat diet

**Fig. 3:**
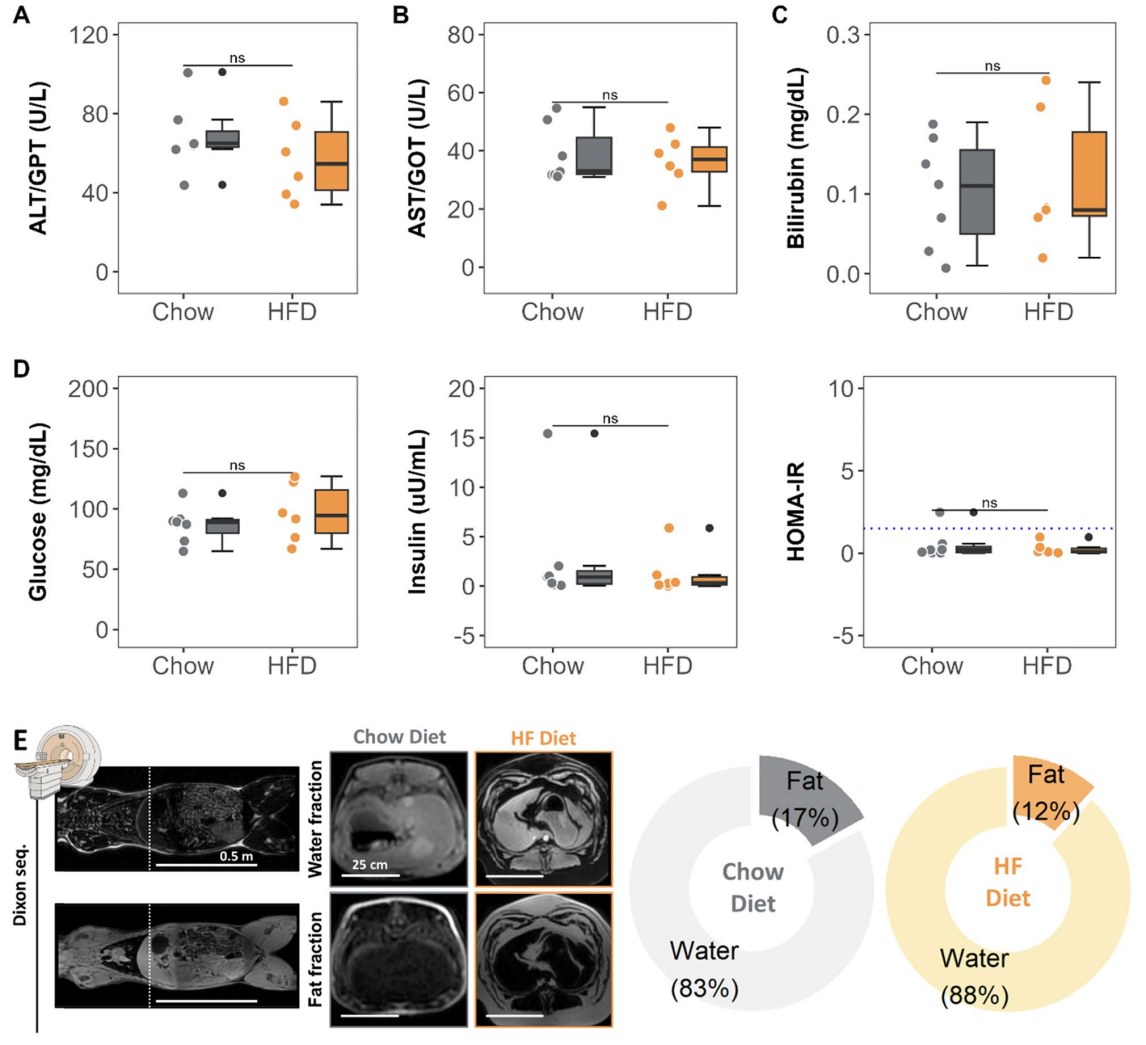
Effect of HF diet on circulating hepatic enzymes, insulin resistance and composition. **A–C** Circulating plasma liver metabolism parameters. **D** Circulating plasma levels of glucose, insulin and Homeostatic Model Assessment of Insulin Resistance (HOMA-IR) index. **E** Representative Dixon magnetic resonance images and relative liver water and fat composition. Colored dots and lines indicate individual values for each group (gray for Chow diet and orange for HF diet). Tukey’s boxplots show median values (bold lines), interquantile range (boxes), and the remaining data distribution (whiskers). Asterisks indicate statistical significance as follows: ns: no significance, *p < 0.05, **p < 0.01, ***p < 0.001. (non-parametric Wilcoxon rank test). Sample size: n = 7 in Chow and n = 6 in HFD group. Scale bars for each imaging are presented.

The 2-month HFD caused a significant increase in total plasma cholesterol at the expense of increased HDL-cholesterol. Triglycerides, LDL-cholesterol, liver enzymes, and insulin resistance index (HOMA-IR) were not different between groups at the end of the 2 months intervention (Figs. 2 and 3). Dixon magnetic resonance imaging revealed no difference in hepatic fat infiltration between the HFD and control diet groups (Fig. 3).

### High-fat diet improves cardiac function after heart failure secondary to myocardial dysfunction

Baseline and final CMR parameters are shown in Table 1. The randomization procedure was designed to ensure no between-group difference in baseline LVEF (HFD, 38% [33.3, 43.5], Chow, 36% [35.5, 44.8]; *p* = 0.943). There were no baseline between-group differences in LV end-diastolic and LV end-systolic volumes (LVEDV and LVESV), LV mass, and cardiac output.

**Table 1:** CMR parameters and baseline and at the end of the study

	Baseline			2 months				
	Chow (*N* = 7)	HFD (*N* = 6)	P-value Chow vs HFD	Chow (*N* = 7)	HFD (*N* = 6)	*P*-value Chow vs HFD	*P*-value Chow Paired vs Basal	*P*-value HFD Paired vs Basal
Body weight (kg)	69.0 [58.5, 73.0]	66.3 [58.3, 72.0]	0.886	94.5 [80.0, 104]	89.8 [83.3, 111]	0.628	**0.022**	**0.031**
LV Mass (g/m^2^)	72.6 [66.5, 78.5]	76.2 [64.9, 89.5]	0.628	78.6 [73.4, 81.8]	64.3 [54.2, 84.8]	0.445	0.16	0.31
LVEDV (mL/m^2^)	117 [112, 133]	103 [79.1, 144]	0.445	130 [118, 134]	113 [93.8, 146]	0.534	0.47	0.84
LVESV (mL/m^2^)	70.4 [59.7, 80.9]	62.3 [47.6, 89.9]	0.945	69.2 [66.1, 77.9]	54.4 [38.6, 82.0]	0.628	0.94	0.063
LVEF (%)	36.0 [35.5, 44.8]	38.0 [33.3, 43.5]	0.943	41.0 [38.0, 43.3]	54.0 [47.0, 62.5]	**0.012**	0.24	**0.036**
CO (L/min/m^2^)	3.54 [3.08, 4.37]	3.39 [2.87, 4.11]	0.534	3.42 [3.38, 4.04]	4.09 [3.34, 4.61]	0.628	0.94	0.16
Wall thickening anterior (%)	19.5 [16.8, 45.5]	20.0 [14.4, 27.9]	0.567	23.5 [19.3, 44.3]	57.3 [51.0, 66.1]	**0.005**	0.81	**0.036**
Wall thickening posterior (%)	48.0 [43.3, 55.8]	46.0 [39.9, 57.4]	0.945	39.0 [31.5, 52.3]	72.3 [68.9, 76.8]	**0.003**	0.38	**0.094**
LGE (%LV)	9.79 [2.47, 9.98]	2.43 [1.49, 4.65]	0.534	6.77 [5.59, 11.3]	0.455 [0.165, 1.67]	**0.0047**	0.58	**0.094**
LGE transmurality (%, anterior)	13.9 [3.97, 23.8]	4.57 [2.25, 7.86]	0.234	13.1 [9.19, 24.2]	1.6 [0.935, 4.36]	**0.022**	1	**0.22**

LV, left ventricular; LVEDV, LV end-diastolic volume; LVESV, LV end-systolic volume; LVEF, LV ejection fraction; CO, cardiac output; LGE, late gadolinium enhancement. Wilcoxon non-parametric test was used (paired for 2 m-basal in each group and non-paired for groups comparisons)

Data are expressed as median [Q1, Q3]

*p* < 0.05 are represented as bold characters

**Fig. 4:**
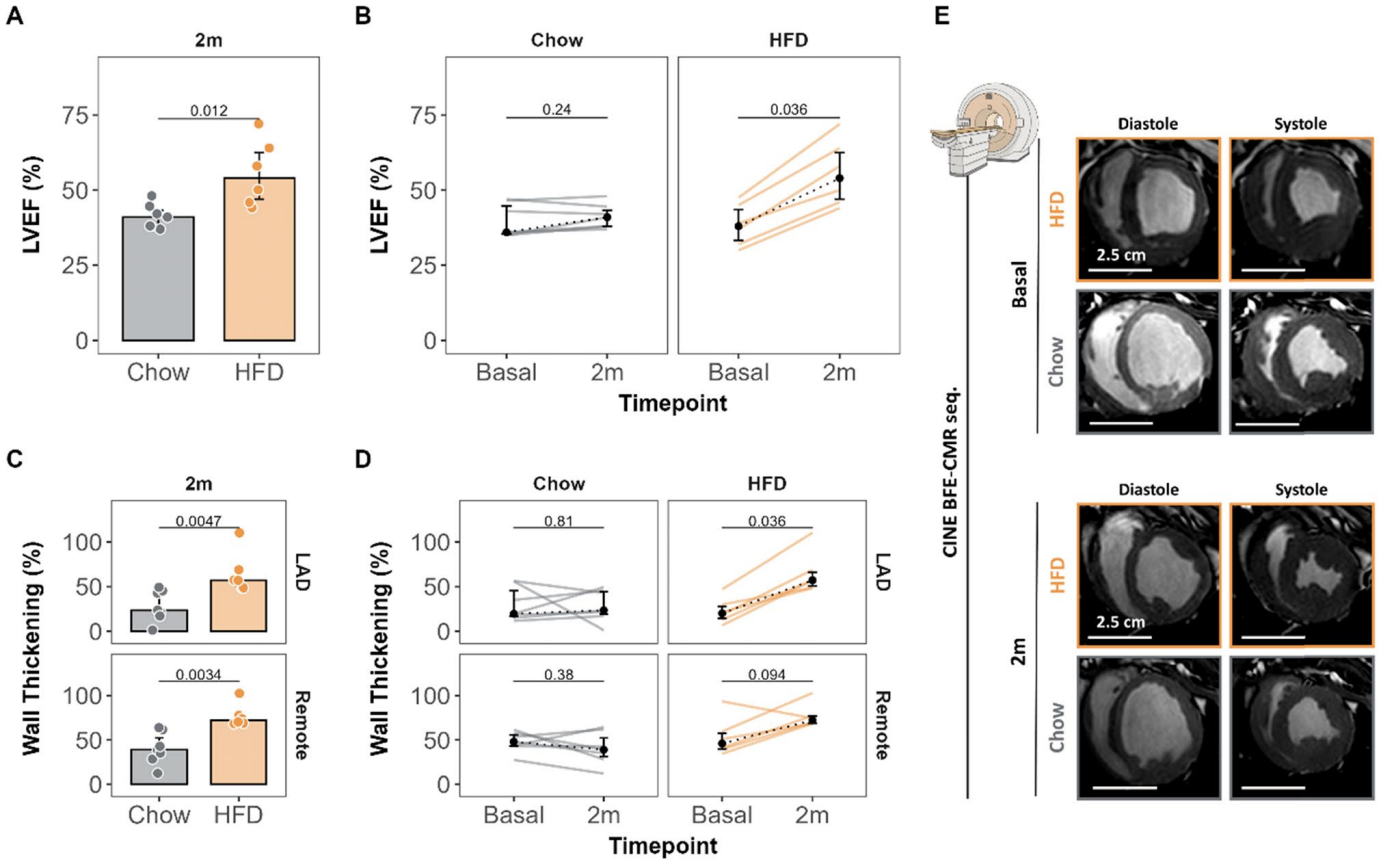
Restoration of cardiac function and contractility in pigs fed a high-fat diet for 2 months. **A–B** Comparison of LVEF (%) between groups (**A**) and timepoints (**B**). **C–D** Comparison of wall thickening (%) in the mid-LAD region between groups (**C**) and timepoints (**D**). **E** Representative CMR CINE sequences in systole and diastole (mid-ventricular short-axis view) for each group at each timepoint. Colored dots and lines indicate individual values for each group (gray for Chow diet and orange for HF diet). Barplots show median values, and error bars (interquartile range). For timepoint plots, point and error bars represent median and interquartile range, and dotted lines plot the change in median value from before to after the dietary intervention (non-parametric Wilcoxon rank test, paired for timepoint comparison in each group). Sample size: n = 7 in Chow and n = 6 in HFD group. Scale bars for each imaging are presented. BFE-CMR seq, balance turbo fill echo-cardiac magnetic resonance sequence; HFD, high-fat diet; LVEF, left ventricular ejection fraction

After the 2-month nutritional intervention, LVEF significantly improved in HFD-fed pigs (from 38% [33.3, 43.5] to 54% [47.0, 62.5], *p* = 0.036) but remained unchanged in control-diet pigs (36% [35.5, 44.8] to 41% [38.0, 43.3], *p* = 0.24). End-of-intervention LVEF (the primary outcome measure of the study) was significantly higher in HFD-fed pigs (54.0% [47.0, 62.5] vs 41.0% [38.0, 43.3] in control-diet pigs, *p* = 0.012) (Fig. 4A-B).

To study whether the improved systolic function was secondary to increased contractile capacity in the LAD area or was driven by hypercontraction in the remote region, we measured regional systolic function. Wall thickening in the anterior region significantly improved in HFD-treated pigs (20% [14.4, 27.9] to 57.3% [51.0, 66.1], *p* = 0.036) but was unchanged in control-diet pigs (19.5% [16.8, 45.5] to 23.5% [19.3, 44.3], *p* = 81) (Fig. 4C-D). At the end of the study, LAD-region wall thickening was significantly more pronounced in HFD-fed pigs than in animals fed the control diet (57.3% vs 23.5%, *p* = 0.0047). Similar almost significant trend (*p* = 0.094) improvement in contractility was found in remote myocardium of HFD-fed pigs (Fig. 4D).

HFD-fed pigs also showed a non-significant trend toward increased cardiac output that was not observed in control-diet pigs (Table 1). Neither of the study groups showed significant changes in LV mass or LV volume between baseline and the end of the dietary intervention (Table 1).

### High-fat diet nutritional intervention reduces myocardial fibrosis in the failing heart

**Fig. 5:**
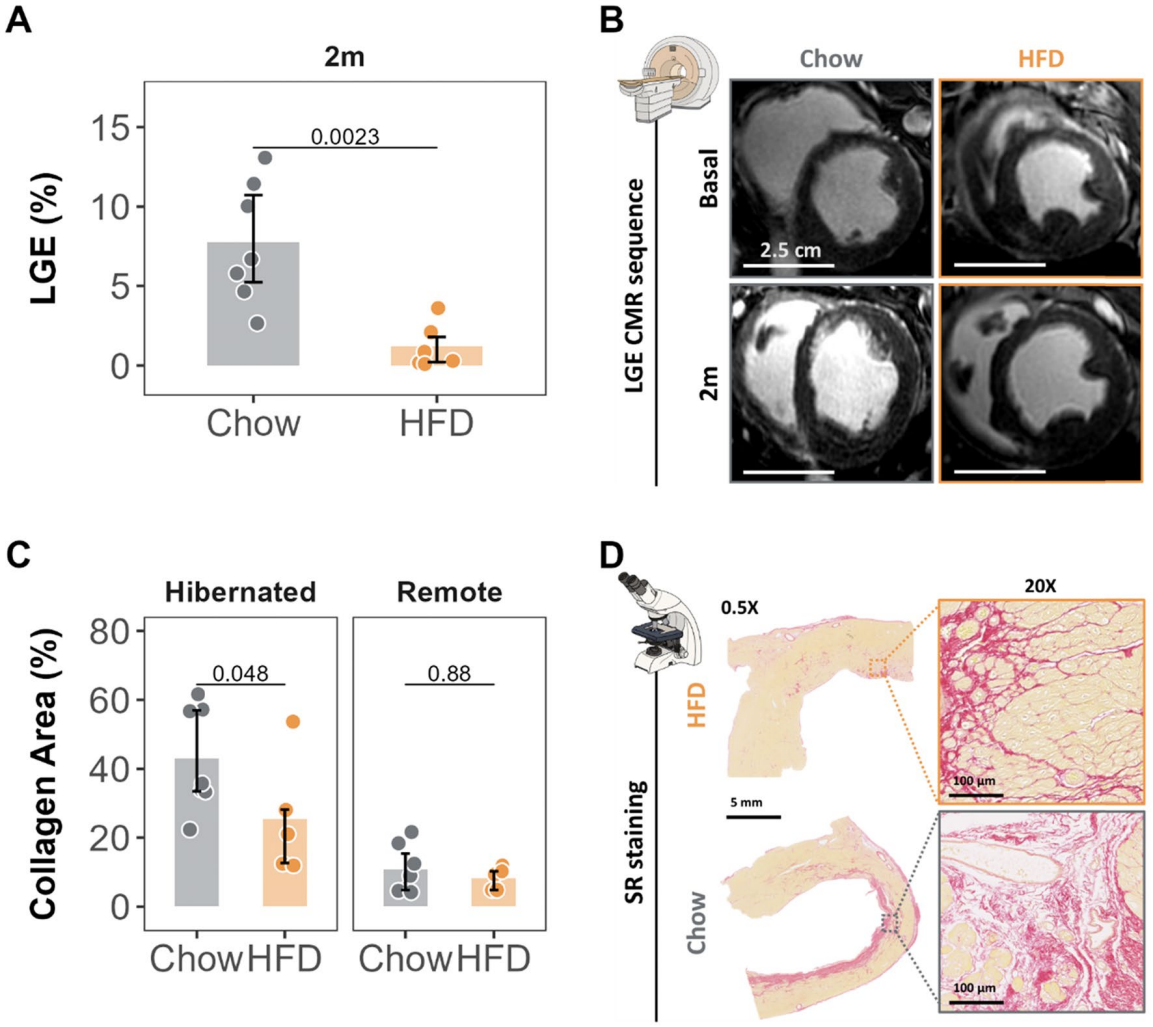
Myocardial fibrosis before and after HFD intervention. **A** Comparison of the extent of LGE (% of LV) between groups at 2 months. **B** Representative CMR LGE sequences (after 3D-segmentation in the short axis) for each group at each timepoint. **C** Collagen fraction area (%) in the myocardium at 2 months in the LAD (anterior) and Cx (remote) irrigated areas, showing comparison between groups. **D** Representative Sirius-Red staining in the LAD region in each group (5 mm scale bar for 0.5 × and 100 μm for 20 × magnifications). Colored dots and lines indicate individual values for each group (gray for Chow diet and orange for HF diet). Bars represent median and error bars interquartile range (non-parametric Wilcoxon rank test). Sample size: *n* = 7 in Chow and *n* = 6 in HFD group. Scale bars for each imaging are presented. HFD, high-fat diet; LGE-CMR, late gadolinium enhancement cardiac magnetic resonance; SR, sirius red

The predefined criteria for the potentially viable myocardium phenotype required the absence of transmural LGE, a surrogate measure of fibrosis. Thus, at baseline the extent of LGE was low and was restricted to the subendocardial myocardium (4.58% LV [2.03, 9.39] for the pooled study groups). At the end of the 2-month nutritional intervention, pigs receiving the HFD had significantly less extensive LGE than control-diet pigs (0.455% LV [0.165, 1.67] vs 6.23% LV [5.54, 9.57], *p* = 0.0043) (Fig. 5A-B**)**. Moreover, in the HFD group, the extent of LGE declined significantly between the baseline and final CMR examinations (2.43% LV [1.49, 4.65] vs 0.455% LV [0.165, 1.67], *p* = 0.031). Baseline and final LGE values in each group are shown in Table 1. These results were similar regarding the transmurality of the contrast deposition as well (Table 1).

Consistent with the CMR data, Sirius-red staining of cardiac samples showed that the collagen-fraction area in the LAD region was significantly smaller in HFD-fed pigs than in their chow-fed counterparts (21.1% [12.6, 28.2] vs 36.0% [33.5, 57.0], *p* = 0.048). No between-group differences in collagen density were found in the remote regions (Fig. 5C–D). 

### A 2-month high-fat diet restores preferential cardiac fatty acid metabolism

**Fig. 6:**
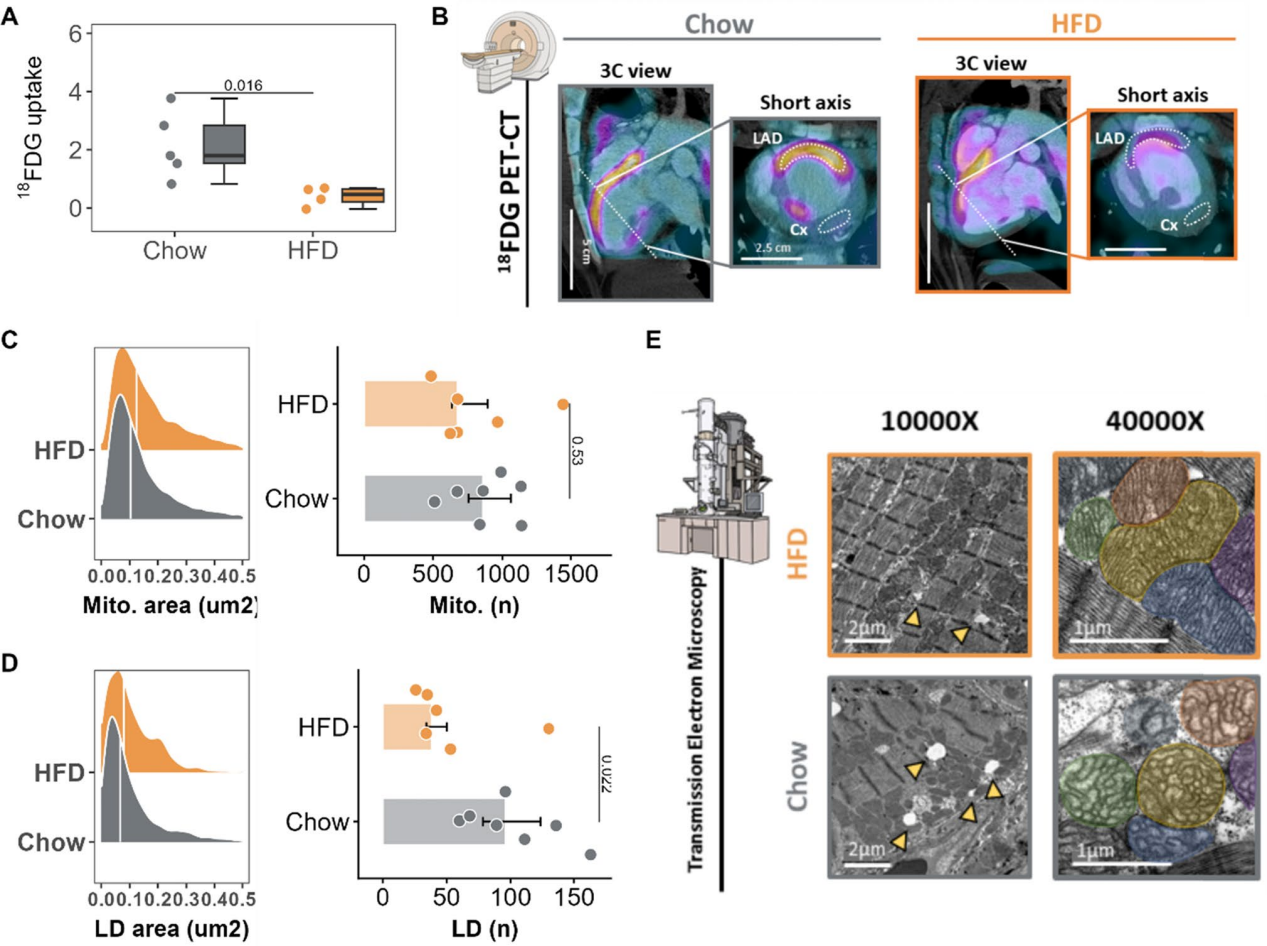
In vivo positron emission tomography scan and transmission electron microscopy analysis after 2 months of high-fat diet. **A** End-of-study ^18^FDG PET count ratio (LAD/Cx irrigated regions) normalized to the blood pool. **B** Representative end-of-study ^18^FDG PET images in 3-chamber and short-axis views. Regions of interest (white dotted lines) are placed around the LAD- and Cx-irrigated regions. **C** Mitochondrial area distribution with median values indicated by vertical white lines (left) and mitochondrial number (right). **D** Lipid droplet area distribution with median values indicated by vertical white lines (left) and lipid droplet number (right). **E** Representative cardiac TEM images from HFD and Chow-diet pigs. Yellow arrows indicate lipid droplet deposition (scale bar of 2 μm for 10000X and 1 μm for 40000X). Mitochondria are colored in the 40,000× magnification view to illustrate between-group differences in mitochondrial area. In panels A, C, and D, colored dots indicate individual values (gray for Chow diet and orange for HFD). The Tukey boxplot in A shows median values (bold lines), interquantile range (boxes), and the remaining data distribution (whiskers). Bars represent median and error bars interquantile range (non-parametric Wilcoxon rank test). Sample size: *n* = 7 in Chow and *n* = 6 in HFD group. Scale bars for each imaging are presented. 18FDG-PET: 18 Fluor-Fluorodeoxyglucose; HFD: high-fat diet

**Fig. 7:**
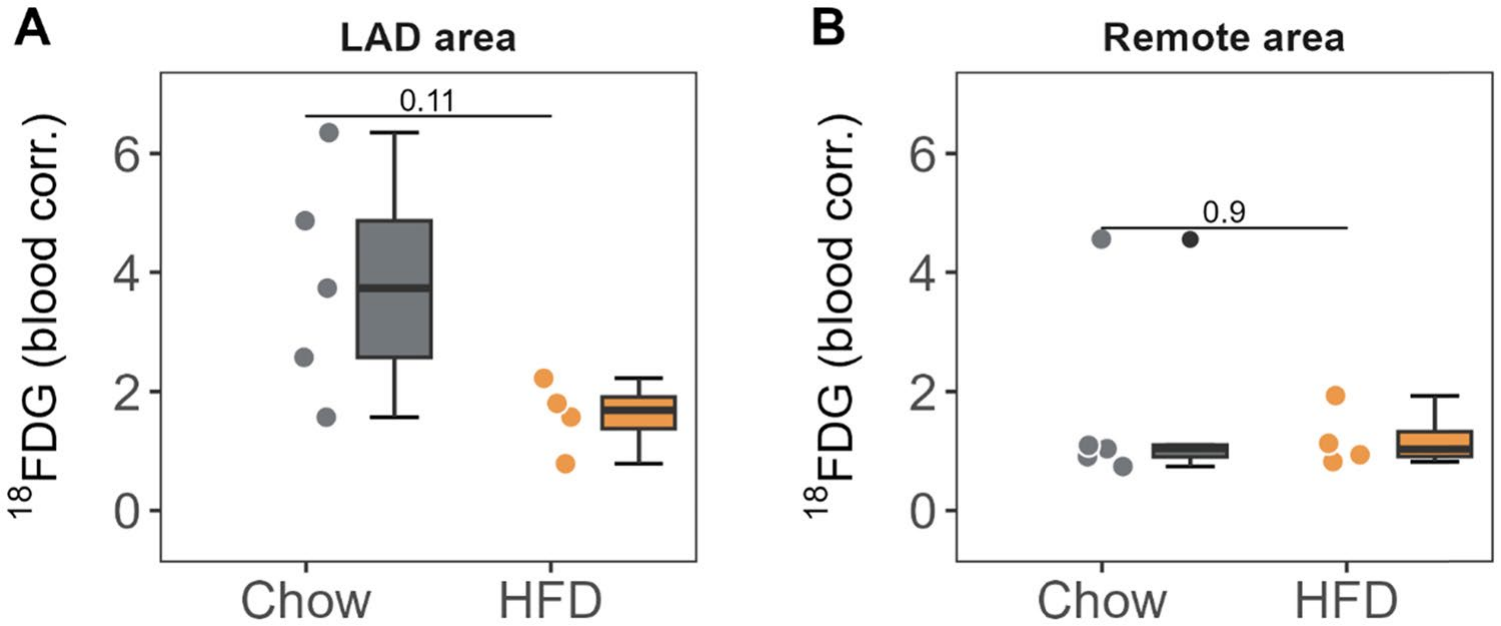
Myocardial FDG uptake normalized by blood pool in LAD-hypoperfused and remote regions. **A** TBR in the LAD and **B** remote regions. Colored dots and lines indicate individual values for each group (gray for Chow diet and orange for HF diet). Tukey’s boxplots show median values (bold lines), interquantile range (boxes), and the remaining data distribution (whiskers). Absolute p-value is displayed. (non-parametric Wilcoxon rank test). Sample size: *n* = 5 in Chow and *n* = 4 in HFD group.

As with most forms of HF, our pig model of HFrEF secondary to myocardial dysfunction features a metabolic switch to glucose metabolism [32]. To study if the HFD-induced improvement in cardiac systolic function was associated with a reversal of this metabolic switch, we examined the pigs by in vivo ^18^FDG-PET/CT at the end of the treatment period. In HFD-fed pigs, glucose uptake in the anterior territory (normalized to the remote region) was significantly lower than that recorded in control-diet pigs (0.46 counts [0.214, 0.646] vs 1.80 [1.53, 2.83], *p* = 0.016) (Fig. 6A-B). No significant changes in glucose metabolism were observed in the remote myocardium in response to the diet, as reflected by comparable TBR values across groups (Fig. 7).

**Fig. 8:**
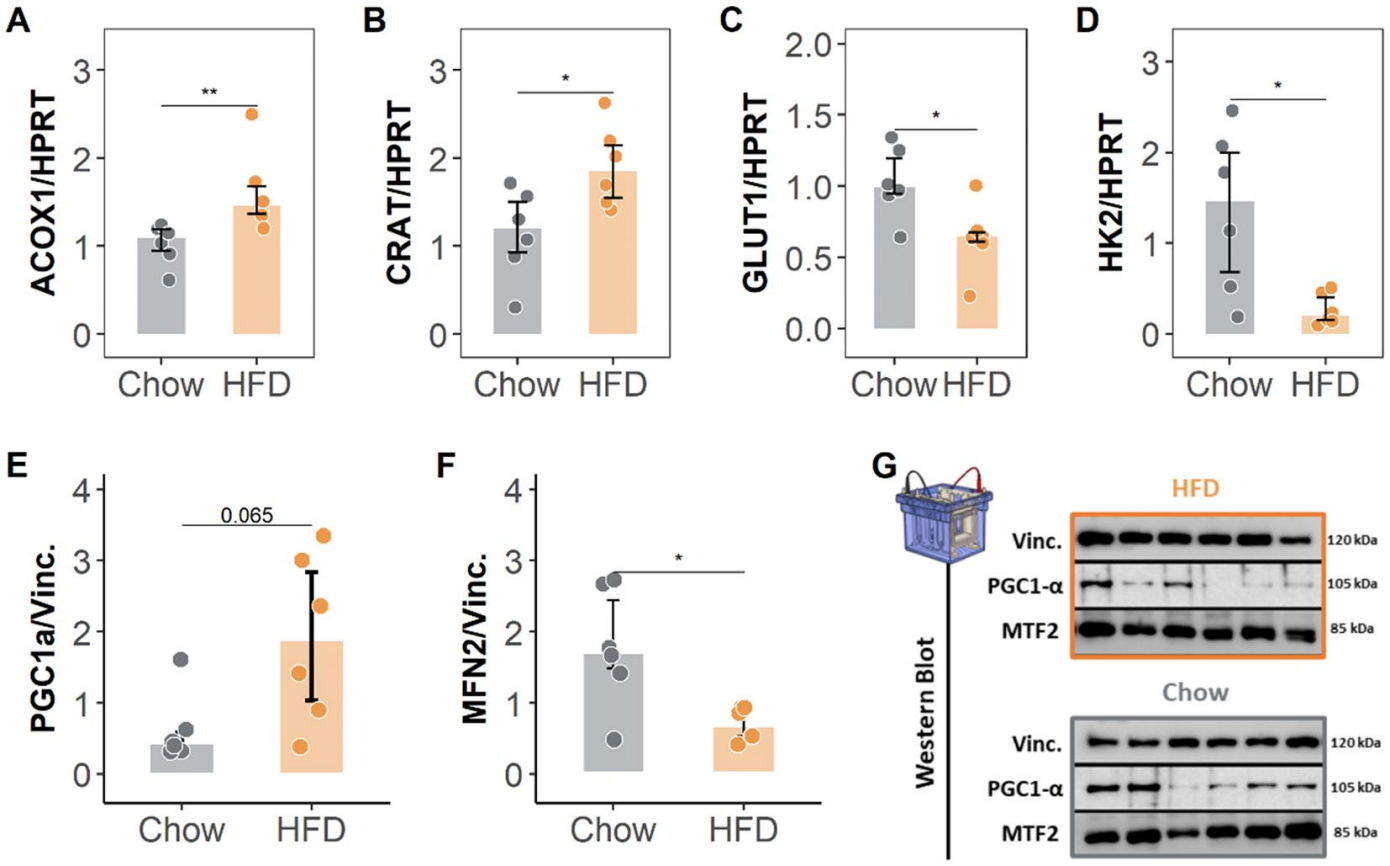
Transcripts and protein expression analyses of cardiac metabolism. Transcripts determination by qPCR and protein expression by western blot. **A–D** End-of-study expression of selected mRNA transcripts involved in lipid metabolism (**A** and **B**) and glucose mobilization **(C** and **D**) normalized to hypoxanthine phosphoribosyltransferase (HPRT). **E–G** End-of-study western blot analysis of the protein expression of (**E**) PGC1α and (**F**) Mitofusin 2, showing the fold change in vinculin-normalized expression. Panel **G** shows the western blots of all samples. Colored dots indicate individual values for each group (gray for Chow diet and orange for HF diet). Bars represent median and error bar interquantile range. Asterisks indicate statistical significance as follows: ns: no significance, *p < 0.05, **p < 0.01, ***p < 0.001. (non-parametric Wilcoxon rank test). Sample size: n = 7 in Chow and n = 6 in HFD group. HFD: high-fat diet

To examine the effect of HFD on cardiac metabolism in more detail, we assessed mitochondrial density and morphology by TEM. Mitochondria in the hearts of HFD-fed pigs were less fragmented (i.e., larger and less numerous) than those in control hearts; however, these differences did not reach statistical significance (Fig. 6C, E). Protein expression of PGC1α (a transcriptional coactivator involved in mitochondrial biogenesis) was upregulated in samples from HFD-fed pigs (1.89-fold [1.03, 2.84] vs 0.434 [0.348, 0.584] in controls, *p* = 0.065). See Fig. 8E, G.

**Fig. 9:**
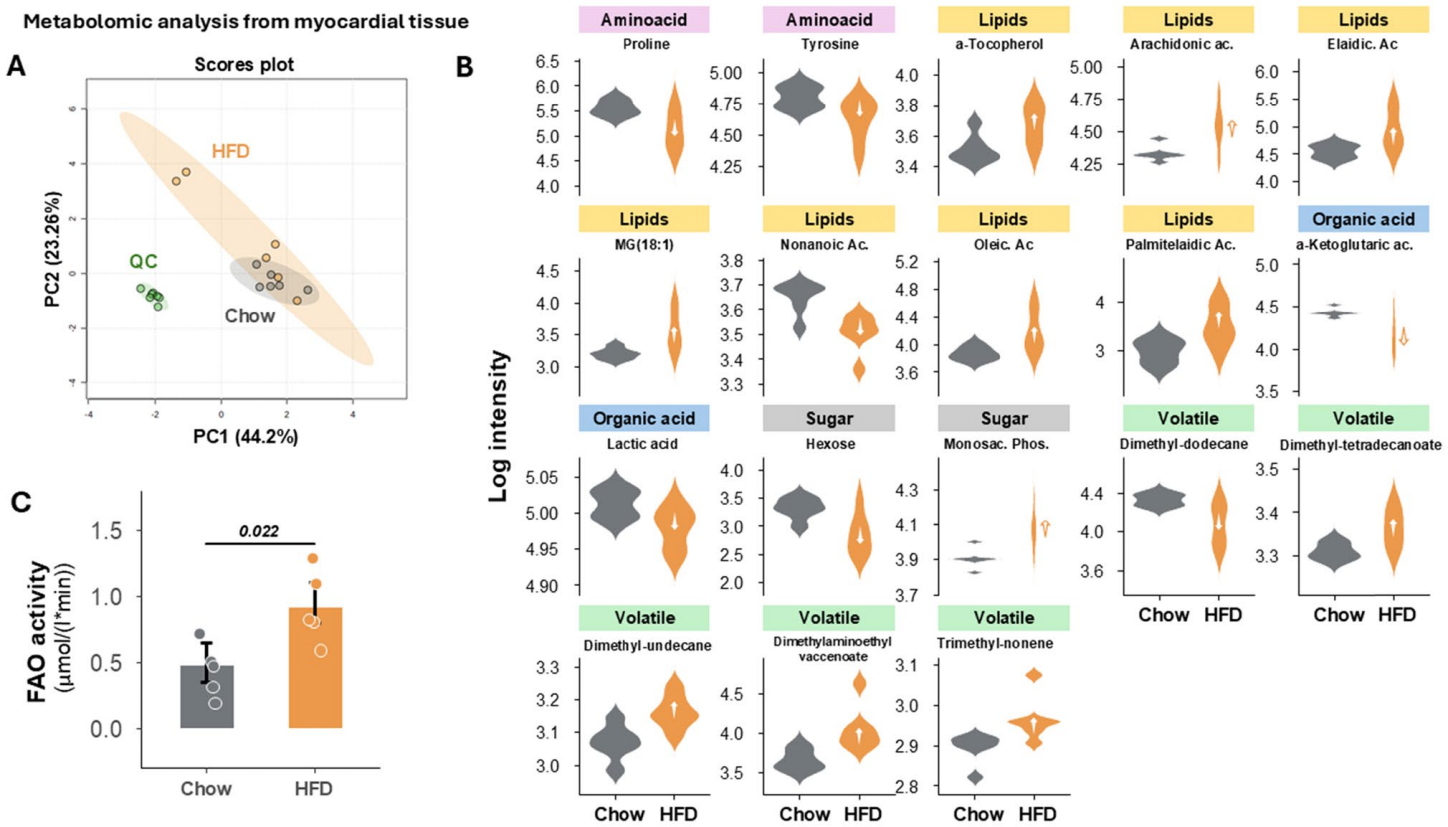
Metabolomics evaluation of myocardial tissue at the end of study. **A** Principal Component Analysis (PCA) score plot for experimental group clustering quality control. **B** Concentration of each metabolite for each category (amino acids, lipids, organic acids, and sugars) and for each group (all statistically significant with p < 0.05). **C** Fatty acid oxidation (FAO) activity for each experimental group. All data at 2 months after the onset of diet; at the end of the study. Violin plots indicate density distribution of the data and arrowed HFD show the effect of the diet on the metabolite concentration (up or down). Bars represent median and error bar interquartile range; colored dots indicate individual values. Asterisks indicate statistical significance as follows: ns: no significance, *p < 0.05, **p < 0.01, ***p < 0.001. (non-parametric Wilcoxon rank test). Sample size: n = 7 in Chow and *n* = 6 in HFD group for metabolomics and *n* = 5 in both groups in FAO assay due to the lack of remaining sample in 3 individuals.

**Fig. 10:**
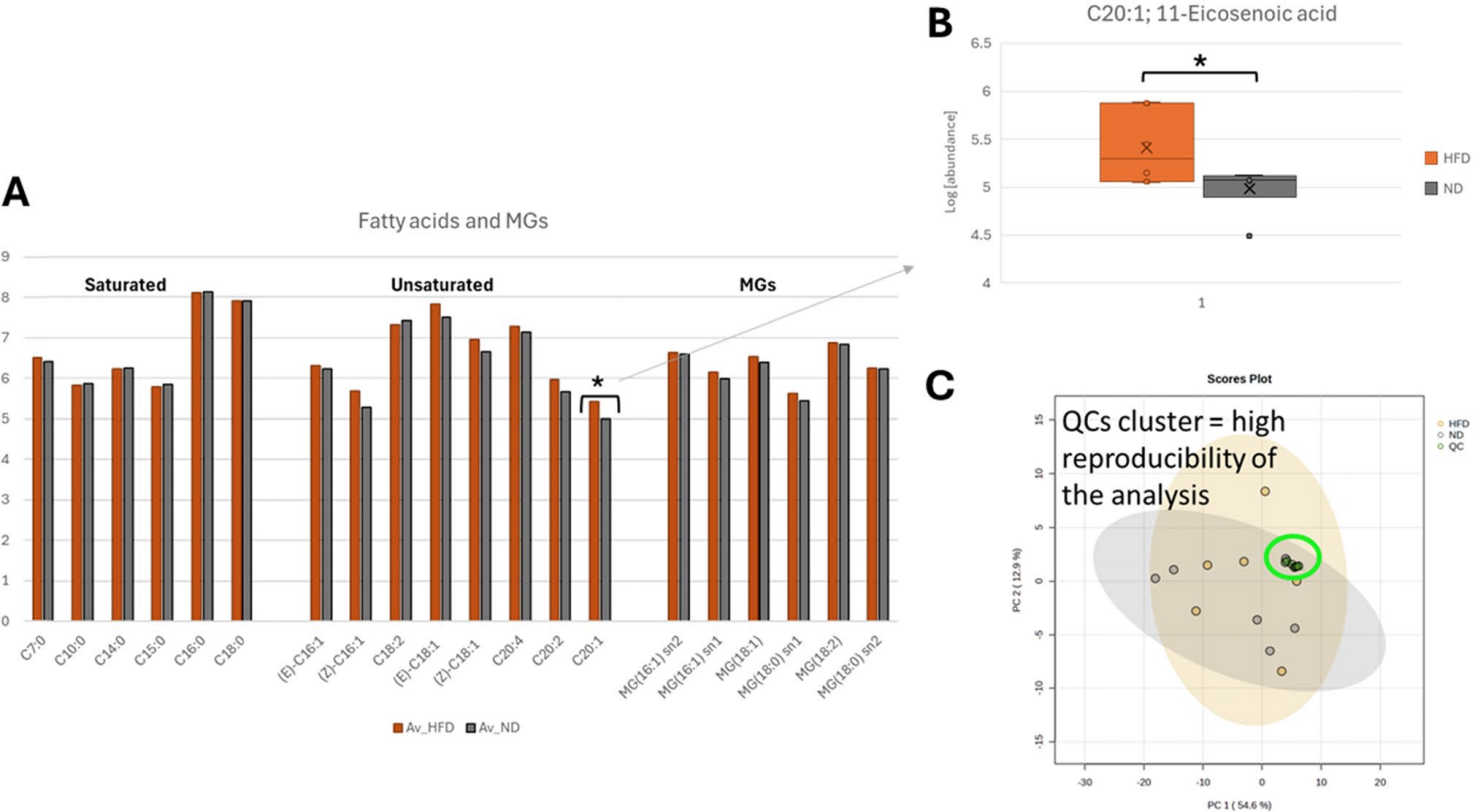
Metabolomic evaluation in the remote tissue. **A** Tissue’s levels of each fatty acid for each group in the remote myocardial area. **B** Concentration of C20:1; 11-Eicosenoic acid, the only one significant. All data at 2 months after the onset of diet; at the end of the study. Boxplots represent median and error bar interquantile range. Asterisks indicate statistical significance as follows: ns: no significance, *p < 0.05, ** *p* < 0.01, ***p < 0.001. (non-parametric Wilcoxon rank test). Sample size: *n* = 7 in Chow (ND) and *n* = 6 in HFD group. **C** Principal Component Analysis (PCA) score plot for experimental group clustering Quality Controls (QCs). MGs: Monoacylglycerols

The hearts of HFD-fed pigs also had a significantly lower lipid-droplet density than hearts from control-diet pigs (38.5 [34.3, 50.3] vs 96.0 [78.5, 124], *p* = 0.022) (Fig. 6D, E). Consistent with this result, protein expression of MMFN2 (a key mediator of lipid-droplet formation) was significantly downregulated in hearts from HFD-fed pigs (0.696-fold [0.532, 0.916] vs 1.72 [1.48, 2.45] in controls, *p* = 0.041) (Fig. 8F, G). Finally, we assessed the expression of key genes involved in fatty acid and glucose intra-cardiomyocyte trafficking. Expression of the key beta-oxidation enzyme ACOX1 was significantly upregulated in hearts from HFD-treated pigs (1.46 [1.37, 1.67] vs 1.09 [0.943, 1.19], *p* = 0.0043). Similarly, hearts from HFD-fed pigs showed significantly upregulated expression of carnitine acetyltransferase (CRAT), a mitochondrial enzyme involved in acetyl-CoA catalyzation (1.85 [1.55, 2.14] vs 1.19 [0.926, 1.50] in controls, *p* = 0.041) (Fig. 8A, B). Conversely, 2 genes involved in glucose mobilization, GLUT1 and HK2, were significantly downregulated in hearts from HFD-fed pigs (0.645 [0.611, 0.678] vs 0.993 [0.946, 1.19], *p* = 0.041; and 0.192 [0.148, 0.400] vs 1.46 [0.680, 2.00], *p* = 0.015, respectively) (Fig. 8C, D). Consistent with these changes, the metabolomic analysis of myocardial tissue revealed significant energetic shifts in pigs treated with HFD. Specifically, glucose was found to be significantly reduced, suggesting decreased reliance on glucose metabolism. Concurrently, lactate levels were lower in the treated group, indicating reduced anaerobic metabolism. Increased levels of free fatty acids and their derivatives, such as oleic acid, arachidonic acid, and monoglycerides, suggest changes in the fatty acid (FA) oxidation. Additionally, alterations in amino acid metabolism, particularly elevated proline levels, point to potential changes in extracellular matrix remodeling (Fig. 9A, B). Moreover, FA oxidation activity was also increased in animals treated with HFD (Fig. 9C). When examining the metabolic effect of dieting in the remote area, we observed that most of the metabolites altered in the LAD stenotic area showed similar directional changes, although they did not reach statistical significance. Specifically, when focusing on the fatty acids and monoacylglycerols, we observed an increased trend in the remote region’s levels as well, with 11-eicosenoic acid being the only metabolite reaching significance (Fig. 10).

Together, these findings suggest that HFD rewires cardiac metabolism to preferential use of fatty acids by restoring myocardial lipid trafficking mainly in the dysfunctional area, with milder effects in the remote myocardium.

## Discussion

Heart failure is characterized by a switch from fatty acids to glucose as the main fuel source for cardiac energy production. Whether this metabolic reprogramming contributes to cardiac dysfunction, and is thus a therapeutic target, remains controversial. In this study, we have examined the impact of a nutritional intervention to increase the proportion of calories obtained from fat in a highly translatable pig model of HFrEF secondary to myocardial dysfunction. Myocardial dysfunction, secondary to altered coronary flow, [28] is an ideal model since it is characterized by severe morphological and metabolic alterations [27]. The main results of this study can be summarized as follows: (1) Two months of HFD (regular chow enriched with 20% lard) resulted in a significant increase in CMR-evaluated LVEF (from 38 to 54%), whereas no change was observed in control animals receiving normal chow. (2) The improvement in systolic function was driven by increased contractility in mainly the viable dysfunctional but also with a mild support from the remote regions. (3) The improved systolic function was associated with a reduction in cardiac fibrosis. (4) PET/CT revealed that the nutritional HFD-based intervention was associated with cardiac metabolic reprogramming also in the dysfunctional region. (5) The intra-cardiomyocyte accumulation of lipid droplets (a typical feature of HFrEF) was alleviated by the 2-month HFD as detected by transmission electron microscopy. (6) The HFD was associated with a significant upregulation of genes involved in cardiac fatty acid trafficking and downregulation of the glucose mobilization machinery. Moreover, changes in metabolite level and increased lipid oxidation activity in HFD-fed pigs also pointed to the preferential use of fatty acids as fuel source. To our knowledge, this is the first study to show a therapeutic effect of a fat-based nutritional intervention in a large animal model resembling human pathophysiology.

### Cardiac metabolic switch in heart failure

Under physiological conditions, the heart derives most of its energy from fatty acid oxidation. Experimental and human studies have consistently shown that HF, independently of its etiology, is associated with a cardiac metabolic alteration involving decreased use of fatty acids and increased glucose uptake and glycolysis [1–3, 9, 21, 25, 29, 40, 56].

Consistent observations of the accumulation of lipid droplets in cardiomyocytes [4] led investigators to propose that the failing heart exists in a state of insulin resistance and fatty acid overload, with the accumulation of cardiotoxic fatty acid derivatives causing oxidative stress [10, 11, 39]. Thus, for many years, reducing cardiac fatty acid use was a therapeutic goal in the management of HF [49]. However, this strategy was later shown to produce no benefits and even to have detrimental effects in patients with end-stage HF, where it significantly worsened LVEF [54]. More recently, evidence from murine models has suggested that a diet rich in fat and low in carbohydrate can prevent the development and progression of HF [6, 49].

### Intervention studies testing a high-fat diet as a nutritional therapy for heart failure

Previous studies undertaken in small animal models of experimental HF have tested the potential benefits of increasing fat intake (HFD or fatty acid supplementation) [10, 38]. These studies used mouse and rat models of LV pressure overload [12, 19, 51, 53]. Our group subsequently showed the benefits of HFD in a mouse model of dilated cardiomyopathy secondary to genetically induced mitochondrial fragmentation [55]. Thus, these murine studies collectively showed that increasing the % of daily calories derived from fat prevents LV hypertrophy and remodeling and mitochondrial fragmentation, alters cardiac metabolite levels, and reduces myocardial fibrosis. Nevertheless, other studies have reported negative effects of HFD; for example, in a mouse model of LV remodeling secondary to pressure overload, HFD induced insulin resistance and had a deleterious effect on LV remodeling [42]. The present study is the first to demonstrate beneficial effects of HFD in a large animal model of HFrEF using serial state-of-the-art imaging methodology. Furthermore, our study tested the benefits of the nutritional intervention after the appearance of overt cardiac dysfunction. This is important because clinical interventions are initiated only after the diagnosis of HFrEF. The preventive effect of HFD reported in earlier murine studies is of less translational value.

Animal studies investigating the effects of a low-carbohydrate HFD need to be evaluated with caution since diet-induced obesity is frequently a confounding factor. This concern may be related to the “obesity paradox” in HF, wherein obese people have double the risk of developing HF of normal-weight individuals [30], but once diagnosed have better survival and lower hospitalization rates than non-obese HF patients [31]. In our study, pigs on HFD did not gain more weight than those on the control diet. Thus, obesity, and the obesity paradox, would not appear to have contributed significantly to the improved cardiac function observed in our study.

### Mechanisms of the HFD-induced improvement in heart function

Emerging evidence suggests that the intra-cardiomyocyte accumulation of lipids in failing conditions leads to an energy deficit rather than to lipotoxicity and signaling imbalance [44–46, 49]. The metabolic switch in HF involves a fetal-like gene reprogramming, with repression of genes encoding key fatty acid oxidation enzymes accompanied by upregulation of genes encoding proteins involved in glucose import [43]. In our large animal study, HF was associated with defective lipid trafficking, evidenced by lipid droplet accumulation and the transcript downregulation of CRAT (responsible for acetyl-CoA catalysis) and ACOX1 (an enzyme involved in the β-oxidation pathway) as well as increased fatty acid oxidation. The 2-month HFD normalized the expression of these genes, and this was associated with a significant reduction in lipid droplet content detected by TEM. We also detected downregulation of MFN2, a protein involved in mitochondrial fusion and in the formation of lipid droplets. These findings, together with the reduced mitochondrial fragmentation (with no impact on the expression of transport chain proteins), suggest a restoration of the homeostatic metabolic state in the myocardium.

These results suggest that HFD normalizes lipid traffic through a yet undefined mechanism. This conclusion is consistent with a previous report that elevated circulating concentrations of fatty acids after HFD increase the fatty acid gradient across the sarcolemma, restoring intracellular lipid flux [18, 41]. Another proposed mechanism for the cardioprotection afforded by a HFD is the induction of insulin resistance [24]. By reducing glucose import to the myocardium, insulin resistance might promote the restoration of fatty acid use as the principal fuel for energy production. However, biochemical analysis in our study showed no evidence of insulin resistance.

In our study, we observed that HFD resulted in an increased contractility of the LAD-hypoperfused region, but also in the remote region despite it being non-significant. The latter is intriguing since this effect occurred despite no increased glucose uptake being observed in the remote region at baseline or after diet intervention in the HFD group. This result might imply that additional mechanisms beyond the reversion of the metabolic switch might partially explain the benefits observed in the present study, being them mainly led through the diet effect in the LAD-dysfunctional area but, importantly, also involving more subtle diet-driven lipid adaptations in the remote myocardium, as suggested by the selective increase in the long-chain monounsaturated fatty acid C20:1 (11-eicosenoic acid) having translational implication of general diet effect in more diffuse or heterogenic myocardial conditions.

The 2-month time-restricted HFD in our analysis is in line with most previous experimental studies. Tan and collaborators [51] compared short-term (8-week) and long-term (16-week) HFD interventions in murine, finding that the cardioprotection afforded by the 8-week diet was attenuated when the HFD was maintained for 16 weeks. Future studies in the pig model should determine if prolonged periods on the HFD result in the loss of the cardioprotective effect or even trigger a rebound effect. This is a critical point from a translational perspective, given the implication that chronic HF patients may not gain any advantage from an unlimited HFD, and it may prove necessary to intersperse periods of time-limited HFD with periods on a normal diet.

### Types of fat used in experimental studies

Several studies showing benefits of a HFD in murine models of HF used diets enriched in long-chain saturated fatty acids [38], and few studies have compared different types of HFD. In a genetic model of cardiomyopathy in hamsters, a diet enriched in saturated fats (palmitate and stearate) improved HF parameters relative to a diet enriched in polyunsaturated fatty acids (PUFA) [15]. In a rat model of hypertensive cardiomyopathy, linoleate-rich and lard-rich diets both attenuated LV hypertrophy, but only the linoleate-rich diet reduced HF-related mortality [7].

In human studies, fatty acids have mostly been administered in capsules, and thus not strictly speaking as a nutritional intervention. In the large GISSI-HF trial, 6900 HF patients were randomized to receive n-3 PUFAs (0.9 g/d) or placebo. While HF admission was not reduced, patients in the fatty acid supplement arm had lower all-cause mortality [52]. In a much smaller trial, a 2 g/d dose of n-3 PUFAs was associated with a 10% increase in LVEF [37]. The benefits of n-3 PUFAs administered in capsules seem to be dose dependent [35]. A few studies have examined the effect of diets enriched in polyunsaturated fatty acids (including fish oils), but these were undertaken in small samples of fewer than 15 patients [5, 34].

The HFD in our study was regular chow supplemented with 20% lard. While this proportion is low compared with the 40% fat-derived calories in a balanced human diet, it is much higher than the 2% of energy supplied by fat in a regular porcine diet and thus represents a significant change in macronutrient composition. Lard consists of approximately 40% saturated, 45% monounsaturated, and 15% polyunsaturated fatty acids, with the most abundant fatty acids being palmitic and stearic acids (both saturated), oleic acid (monounsaturated), and linoleic acid (polyunsaturated). Our study thus does not address the possibility that the benefits of the HFD are due to a specific fatty acid.

### Epidemiological data related to the effect of fat-rich diets on health

Classic dietary guidance includes recommendations to reduce fat intake. However, in recent years the basis for these recommendations has been challenged [16, 23]. There is now increasing evidence that fat consumption is not associated with cardiovascular disease [48] and that cholesterol in obese adults can be better controlled by restricting the intake of carbohydrates rather than lipids [17]. Moreover, epidemiological studies suggest that diets in which fats represent a high proportion of calorie intake not only are not deleterious but are beneficial for overall health [22, 36]. The large observational PURE study of 135,000 individuals [8] demonstrated links between lower total mortality and the intake of total fat and individual types of fat. This study even found an inverse association between saturated fat intake and stroke. The primary prevention PREDIMED trial demonstrated that a diet enriched in fats derived from extra-virgin olive oil and nuts was associated with a reduction in the incidence of major cardiovascular events [13]. Therefore, replacing dietary carbohydrate with fat to prevent HF should not be seen as a counterintuitive measure.

Despite these recent advances, current clinical practice guidelines for HF provide no specific recommendations on fat intake and recommend dietary patterns aimed at reducing blood pressure without mentioning the potential benefits of dietary fat [26, 33]. The idea of developing optimal diets for the prevention and treatment of HF is particularly attractive, as any beneficial effects should complement or even work in synergy with current treatments with drugs and devices.

## Conclusions

This study in a highly translatable pig model of HFrEF provides evidence for the therapeutic benefits of a nutritional intervention based on HFD. In pigs with overt HFrEF secondary to myocardial dysfunction, a 2-month HFD was associated with cardiac metabolic reprogramming back to the use of fatty acids as the main substrate for energy production, accompanied by a very notable increase in LVEF (15 points) and a reduction in cardiac fibrosis. This study sets the basis for a future pilot clinical trial of a nutritional intervention based on increased dietary fat caloric intake to improve cardiac function in patients with HF.

### Study limitations

The model of viable dysfunctional myocardium used in this study resembles many features of clinical HF; however, it is very costly and lengthy. Since we have characterized this model in the past, including CMR and PET before and after ameroid implant [32], in the present trial, we did not obtain these data before/after surgical ameroid implant. Although the sample size was calculated from a previous pilot study and adjusted to our primary outcome measure, our study includes relatively few animals. To compensate for this, these animals were serially assessed with a comprehensive multimodality imaging protocol. Since we focus the main primary variables on imaging parameters, this study also lacks some hemodynamic parameters. At baseline, the extent of delayed gadolinium enhancement, while not transmural, was numerically (not significantly) larger in control pigs. Heart rate and blood pressure were not recorded paired with imaging studies. Although the increase in cardiac output in HFD-fed animals did not reach statistical significance, it followed the same direction as the improvement in LVEF. This discrepancy is likely due to variability between individual animals, which is not fully captured by the median [Q1, Q3] summary statistics. Nonetheless, both parameters point toward improved cardiac performance, supporting the consistency and robustness of our interpretation. The nature of our study does not allow us to determine whether the benefits observed were driven by the effects of a specific fatty acid or if any source of fat would have had the same therapeutic effect. The trial phase was limited to 2 months, and it is thus unknown if the benefits would have been attenuated after a longer dietary intervention. Similarly, we do not know whether stopping the HFD would result in a relapse of cardiac systolic dysfunction or if metabolic memory would maintain the benefits after a return to a normal diet.

## Data Availability

Data is available upon reasonable request to the corresponding author.

## References

[1] Amorim PA, Nguyen TD, Shingu Y, Schwarzer M, Mohr FW, Schrepper A, Doenst T (2010) Myocardial infarction in rats causes partial impairment in insulin response associated with reduced fatty acid oxidation and mitochondrial gene expression. J Thorac Cardiovasc Surg 140:1160–1167. 10.1016/j.jtcvs.2010.08.003 20850803 10.1016/j.jtcvs.2010.08.003

[2] Beer M, Seyfarth T, Sandstede J, Landschutz W, Lipke C, Kostler H, von Kienlin M, Harre K, Hahn D, Neubauer S (2002) Absolute concentrations of high-energy phosphate metabolites in normal, hypertrophied, and failing human myocardium measured noninvasively with (31)P-SLOOP magnetic resonance spectroscopy. J Am Coll Cardiol 40:1267–1274. 10.1016/s0735-1097(02)02160-5 12383574 10.1016/s0735-1097(02)02160-5

[3] Bugger H, Schwarzer M, Chen D, Schrepper A, Amorim PA, Schoepe M, Nguyen TD, Mohr FW, Khalimonchuk O, Weimer BC, Doenst T (2010) Proteomic remodelling of mitochondrial oxidative pathways in pressure overload-induced heart failure. Cardiovasc Res 85:376–384. 10.1093/cvr/cvp344 19843514 10.1093/cvr/cvp344

[4] Capone F, Vettor R, Schiattarella GG (2023) Cardiometabolic HFpEF: NASH of the heart. Circulation 147:451–453. 10.1161/CIRCULATIONAHA.122.062874 36745698 10.1161/CIRCULATIONAHA.122.062874

[5] Carbone S, Billingsley HE, Canada JM, Kadariya D, Medina de Chazal H, Rotelli B, Potere N, Paudel B, Markley R, Dixon DL, Trankle CR, Van Tassell BW, Celi FS, Abbate A (2019) Unsaturated Fatty Acids to Improve Cardiorespiratory Fitness in Patients With Obesity and HFpEF: The UFA-Preserved Pilot Study. JACC Basic Transl Sci 4:563–565. 10.1016/j.jacbts.2019.04.001 31468011 10.1016/j.jacbts.2019.04.001 PMC6712039

[6] Chess DJ, Stanley WC (2008) Role of diet and fuel overabundance in the development and progression of heart failure. Cardiovasc Res 79:269–278. 10.1093/cvr/cvn074 18343896 10.1093/cvr/cvn074

[7] Chicco AJ, Sparagna GC, McCune SA, Johnson CA, Murphy RC, Bolden DA, Rees ML, Gardner RT, Moore RL (2008) Linoleate-rich high-fat diet decreases mortality in hypertensive heart failure rats compared with lard and low-fat diets. Hypertension 52:549–555. 10.1161/HYPERTENSIONAHA.108.114264 18663155 10.1161/HYPERTENSIONAHA.108.114264 PMC2864132

[8] Dehghan M, Mente A, Zhang X, Swaminathan S, Li W, Mohan V, Iqbal R, Kumar R, Wentzel-Viljoen E, Rosengren A, Amma LI, Avezum A, Chifamba J, Diaz R, Khatib R, Lear S, Lopez-Jaramillo P, Liu X, Gupta R, Mohammadifard N, Gao N, Oguz A, Ramli AS, Seron P, Sun Y, Szuba A, Tsolekile L, Wielgosz A, Yusuf R, Hussein Yusufali A, Teo KK, Rangarajan S, Dagenais G, Bangdiwala SI, Islam S, Anand SS, Yusuf S, Prospective Urban Rural Epidemiology study i (2017) Associations of fats and carbohydrate intake with cardiovascular disease and mortality in 18 countries from five continents (PURE): a prospective cohort study. Lancet 390:2050–2062. 10.1016/S0140-6736(17)32252-3 28864332 10.1016/S0140-6736(17)32252-3

[9] Dodd MS, Ball DR, Schroeder MA, Le Page LM, Atherton HJ, Heather LC, Seymour AM, Ashrafian H, Watkins H, Clarke K, Tyler DJ (2012) In vivo alterations in cardiac metabolism and function in the spontaneously hypertensive rat heart. Cardiovasc Res 95:69–76. 10.1093/cvr/cvs164 22593200 10.1093/cvr/cvs164 PMC4617603

[10] Doenst T, Nguyen TD, Abel ED (2013) Cardiac metabolism in heart failure: implications beyond ATP production. Circ Res 113:709–724. 10.1161/CIRCRESAHA.113.300376 23989714 10.1161/CIRCRESAHA.113.300376 PMC3896379

[11] Drosatos K, Schulze PC (2013) Cardiac lipotoxicity: molecular pathways and therapeutic implications. Curr Heart Fail Rep 10:109–121. 10.1007/s11897-013-0133-0 23508767 10.1007/s11897-013-0133-0 PMC3647019

[12] Duda MK, O’Shea KM, Lei B, Barrows BR, Azimzadeh AM, McElfresh TE, Hoit BD, Kop WJ, Stanley WC (2008) Low-carbohydrate/high-fat diet attenuates pressure overload-induced ventricular remodeling and dysfunction. J Card Fail 14:327–335. 10.1016/j.cardfail.2007.11.003 18474346 10.1016/j.cardfail.2007.11.003 PMC2702243

[13] Estruch R, Ros E, Salas-Salvado J, Covas MI, Corella D, Aros F, Gomez-Gracia E, Ruiz-Gutierrez V, Fiol M, Lapetra J, Lamuela-Raventos RM, Serra-Majem L, Pinto X, Basora J, Munoz MA, Sorli JV, Martinez JA, Fito M, Gea A, Hernan MA, Martinez-Gonzalez MA, Investigators PS (2018) Primary Prevention of Cardiovascular Disease with a Mediterranean Diet Supplemented with Extra-Virgin Olive Oil or Nuts. N Engl J Med 378:e34. 10.1056/NEJMoa1800389 29897866 10.1056/NEJMoa1800389

[14] Galan-Arriola C, Villena-Gutierrez R, Higuero-Verdejo MI, Diaz-Rengifo IA, Pizarro G, Lopez GJ, Molina-Iracheta A, Perez-Martinez C, Garcia RD, Gonzalez-Calle D, Lobo M, Sanchez PL, Oliver E, Cordoba R, Fuster V, Sanchez-Gonzalez J, Ibanez B (2021) Remote ischaemic preconditioning ameliorates anthracycline-induced cardiotoxicity and preserves mitochondrial integrity. Cardiovasc Res 117:1132–1143. 10.1093/cvr/cvaa181 32597960 10.1093/cvr/cvaa181 PMC7983009

[15] Galvao TF, Brown BH, Hecker PA, O’Connell KA, O’Shea KM, Sabbah HN, Rastogi S, Daneault C, Des Rosiers C, Stanley WC (2012) High intake of saturated fat, but not polyunsaturated fat, improves survival in heart failure despite persistent mitochondrial defects. Cardiovasc Res 93:24–32. 10.1093/cvr/cvr258 21960686 10.1093/cvr/cvr258 PMC3243037

[16] Gardner CD, Vadiveloo MK, Petersen KS, Anderson CAM, Springfield S, Van Horn L, Khera A, Lamendola C, Mayo SM, Joseph JJ, American Heart Association Council on L, Cardiometabolic H, Council on C, Stroke N, Council on H, Council on Peripheral Vascular D (2023) Popular dietary patterns: alignment with American Heart Association 2021 dietary guidance: a scientific statement from the American Heart Association. Circulation. 10.1161/CIR.0000000000001146 37128940 10.1161/CIR.0000000000001146

[17] Gjuladin-Hellon T, Davies IG, Penson P, Amiri Baghbadorani R (2019) Effects of carbohydrate-restricted diets on low-density lipoprotein cholesterol levels in overweight and obese adults: a systematic review and meta-analysis. Nutr Rev 77:161–180. 10.1093/nutrit/nuy049 30544168 10.1093/nutrit/nuy049

[18] Glatz JF, Luiken JJ, Bonen A (2010) Membrane fatty acid transporters as regulators of lipid metabolism: implications for metabolic disease. Physiol Rev 90:367–417. 10.1152/physrev.00003.2009 20086080 10.1152/physrev.00003.2009

[19] Guo Y, Wang Z, Qin X, Xu J, Hou Z, Yang H, Mao X, Xing W, Li X, Zhang X, Gao F (2018) Enhancing fatty acid utilization ameliorates mitochondrial fragmentation and cardiac dysfunction via rebalancing optic atrophy 1 processing in the failing heart. Cardiovasc Res 114:979–991. 10.1093/cvr/cvy052 29490017 10.1093/cvr/cvy052

[20] Hadi AM, Mouchaers KT, Schalij I, Grunberg K, Meijer GA, Vonk-Noordegraaf A, van der Laarse WJ, Belien JA (2011) Rapid quantification of myocardial fibrosis: a new macro-based automated analysis. Cell Oncol (Dordr) 34:343–354. 10.1007/s13402-011-0035-7 21538025 10.1007/s13402-011-0035-7 PMC3162624

[21] Hahn VS, Petucci C, Kim MS, Bedi KC Jr, Wang H, Mishra S, Koleini N, Yoo EJ, Margulies KB, Arany Z, Kelly DP, Kass DA, Sharma K (2023) Myocardial Metabolomics of Human Heart Failure With Preserved Ejection Fraction. Circulation 147:1147–1161. 10.1161/CIRCULATIONAHA.122.061846 36856044 10.1161/CIRCULATIONAHA.122.061846 PMC11059242

[22] Halton TL, Willett WC, Liu S, Manson JE, Albert CM, Rexrode K, Hu FB (2006) Low-carbohydrate-diet score and the risk of coronary heart disease in women. N Engl J Med 355:1991–2002. 10.1056/NEJMoa055317 17093250 10.1056/NEJMoa055317

[23] Harcombe Z, Baker JS, Cooper SM, Davies B, Sculthorpe N, DiNicolantonio JJ, Grace F (2015) Evidence from randomised controlled trials did not support the introduction of dietary fat guidelines in 1977 and 1983: a systematic review and meta-analysis. Open Heart 2:e000196. 10.1136/openhrt-2014-000196 25685363 10.1136/openhrt-2014-000196 PMC4316589

[24] Harmancey R, Lam TN, Lubrano GM, Guthrie PH, Vela D, Taegtmeyer H (2012) Insulin resistance improves metabolic and contractile efficiency in stressed rat heart. FASEB J 26:3118–3126. 10.1096/fj.12-208991 22611083 10.1096/fj.12-208991 PMC3405268

[25] Heather LC, Cole MA, Lygate CA, Evans RD, Stuckey DJ, Murray AJ, Neubauer S, Clarke K (2006) Fatty acid transporter levels and palmitate oxidation rate correlate with ejection fraction in the infarcted rat heart. Cardiovasc Res 72:430–437. 10.1016/j.cardiores.2006.08.020 17034771 10.1016/j.cardiores.2006.08.020

[26] Heidenreich PA, Bozkurt B, Aguilar D, Allen LA, Byun JJ, Colvin MM, Deswal A, Drazner MH, Dunlay SM, Evers LR, Fang JC, Fedson SE, Fonarow GC, Hayek SS, Hernandez AF, Khazanie P, Kittleson MM, Lee CS, Link MS, Milano CA, Nnacheta LC, Sandhu AT, Stevenson LW, Vardeny O, Vest AR, Yancy CW (2022) 2022 AHA/ACC/HFSA guideline for the management of heart failure: executive summary: a report of the American College of Cardiology/American Heart Association Joint Committee on Clinical Practice Guidelines. J Am Coll Cardiol 79:1757–1780. 10.1016/j.jacc.2021.12.011 35379504 10.1016/j.jacc.2021.12.011

[27] Heusch G (2021) Myocardial stunning and hibernation revisited. Nat Rev. 10.1038/s41569-021-00506-7 10.1038/s41569-021-00506-7 33531698

[28] Heusch G (2022) Coronary blood flow in heart failure: cause, consequence and bystander. Basic Res Cardiol 117:1. 10.1007/s00395-022-00909-8 35024969 10.1007/s00395-022-00909-8 PMC8758654

[29] Kato T, Niizuma S, Inuzuka Y, Kawashima T, Okuda J, Tamaki Y, Iwanaga Y, Narazaki M, Matsuda T, Soga T, Kita T, Kimura T, Shioi T (2010) Analysis of metabolic remodeling in compensated left ventricular hypertrophy and heart failure. Circ Heart Fail 3:420–430. 10.1161/CIRCHEARTFAILURE.109.888479 20176713 10.1161/CIRCHEARTFAILURE.109.888479

[30] Kenchaiah S, Evans JC, Levy D, Wilson PW, Benjamin EJ, Larson MG, Kannel WB, Vasan RS (2002) Obesity and the risk of heart failure. N Engl J Med 347:305–313. 10.1056/NEJMoa020245 12151467 10.1056/NEJMoa020245

[31] Lavie CJ, Milani RV, Ventura HO (2009) Obesity and cardiovascular disease: risk factor, paradox, and impact of weight loss. J Am Coll Cardiol 53:1925–1932. 10.1016/j.jacc.2008.12.068 19460605 10.1016/j.jacc.2008.12.068

[32] Martinez-Milla J, Galan-Arriola C, Carnero M, Cobiella J, Perez-Camargo D, Bautista-Hernandez V, Rigol M, Solanes N, Villena-Gutierrez R, Lobo M, Mateo J, Vilchez-Tschischke JP, Salinas B, Cusso L, Lopez GJ, Fuster V, Desco M, Sanchez-Gonzalez J, Ibanez B (2020) Translational large animal model of hibernating myocardium: characterization by serial multimodal imaging. Basic Res Cardiol 115:33. 10.1007/s00395-020-0788-0 32291522 10.1007/s00395-020-0788-0

[33] McDonagh TA, Metra M, Adamo M, Gardner RS, Baumbach A, Bohm M, Burri H, Butler J, Celutkiene J, Chioncel O, Cleland JGF, Coats AJS, Crespo-Leiro MG, Farmakis D, Gilard M, Heymans S, Hoes AW, Jaarsma T, Jankowska EA, Lainscak M, Lam CSP, Lyon AR, McMurray JJV, Mebazaa A, Mindham R, Muneretto C, Piepoli FM, Price S, Rosano GMC, Ruschitzka F, Skibelund KA, Group ESCSD (2021) 2021 ESC guidelines for the diagnosis and treatment of acute and chronic heart failure. Eur Heart J 42:3599–3726. 10.1093/eurheartj/ehab368 34447992 10.1093/eurheartj/ehab368

[34] Mehra MR, Lavie CJ, Ventura HO, Milani RV (2006) Fish oils produce anti-inflammatory effects and improve body weight in severe heart failure. J Heart Lung Transplant 25:834–838. 10.1016/j.healun.2006.03.005 16818127 10.1016/j.healun.2006.03.005

[35] Moertl D, Hammer A, Steiner S, Hutuleac R, Vonbank K, Berger R (2011) Dose-dependent effects of omega-3-polyunsaturated fatty acids on systolic left ventricular function, endothelial function, and markers of inflammation in chronic heart failure of nonischemic origin: a double-blind, placebo-controlled, 3-arm study. Am Heart J 161:e911-919. 10.1016/j.ahj.2011.02.011 10.1016/j.ahj.2011.02.011 21570522

[36] Mozaffarian D, Appel LJ, Van Horn L (2011) Components of a cardioprotective diet: new insights. Circulation 123:2870–2891. 10.1161/CIRCULATIONAHA.110.968735 21690503 10.1161/CIRCULATIONAHA.110.968735 PMC6261290

[37] Nodari S, Triggiani M, Campia U, Manerba A, Milesi G, Cesana BM, Gheorghiade M, Dei Cas L (2011) Effects of n-3 polyunsaturated fatty acids on left ventricular function and functional capacity in patients with dilated cardiomyopathy. J Am Coll Cardiol 57:870–879. 10.1016/j.jacc.2010.11.017 21215550 10.1016/j.jacc.2010.11.017

[38] Okere IC, Chess DJ, McElfresh TA, Johnson J, Rennison J, Ernsberger P, Hoit BD, Chandler MP, Stanley WC (2005) High-fat diet prevents cardiac hypertrophy and improves contractile function in the hypertensive Dahl salt-sensitive rat. Clin Exp Pharmacol Physiol 32:825–831. 10.1111/j.1440-1681.2005.04272.x 16173943 10.1111/j.1440-1681.2005.04272.x

[39] Opie LH, Knuuti J (2009) The adrenergic-fatty acid load in heart failure. J Am Coll Cardiol 54:1637–1646. 10.1016/j.jacc.2009.07.024 19850204 10.1016/j.jacc.2009.07.024

[40] Osorio JC, Stanley WC, Linke A, Castellari M, Diep QN, Panchal AR, Hintze TH, Lopaschuk GD, Recchia FA (2002) Impaired myocardial fatty acid oxidation and reduced protein expression of retinoid X receptor-alpha in pacing-induced heart failure. Circulation 106:606–612. 10.1161/01.cir.0000023531.22727.c1 12147544 10.1161/01.cir.0000023531.22727.c1

[41] Ouwens DM, Diamant M, Fodor M, Habets DDJ, Pelsers M, El Hasnaoui M, Dang ZC, van den Brom CE, Vlasblom R, Rietdijk A, Boer C, Coort SLM, Glatz JFC, Luiken J (2007) Cardiac contractile dysfunction in insulin-resistant rats fed a high-fat diet is associated with elevated CD36-mediated fatty acid uptake and esterification. Diabetologia 50:1938–1948. 10.1007/s00125-007-0735-8 17639306 10.1007/s00125-007-0735-8 PMC2039861

[42] Raher MJ, Thibault HB, Buys ES, Kuruppu D, Shimizu N, Brownell AL, Blake SL, Rieusset J, Kaneki M, Derumeaux G, Picard MH, Bloch KD, Scherrer-Crosbie M (2008) A short duration of high-fat diet induces insulin resistance and predisposes to adverse left ventricular remodeling after pressure overload. Am J Physiol Heart Circ Physiol 295:H2495-2502. 10.1152/ajpheart.00139.2008 18978196 10.1152/ajpheart.00139.2008 PMC2614531

[43] Sack MN, Rader TA, Park S, Bastin J, McCune SA, Kelly DP (1996) Fatty acid oxidation enzyme gene expression is downregulated in the failing heart. Circulation 94:2837–2842. 10.1161/01.cir.94.11.2837 8941110 10.1161/01.cir.94.11.2837

[44] Schiattarella GG, Alcaide P, Condorelli G, Gillette TG, Heymans S, Jones EAV, Kallikourdis M, Lichtman A, Marelli-Berg F, Shah S, Thorp EB, Hill JA (2022) Immunometabolic mechanisms of heart failure with preserved ejection fraction. Nat Cardiovasc Res 1:211–222. 10.1038/s44161-022-00032-w 35755006 10.1038/s44161-022-00032-w PMC9229992

[45] Schiattarella GG, Altamirano F, Kim SY, Tong D, Ferdous A, Piristine H, Dasgupta S, Wang X, French KM, Villalobos E, Spurgin SB, Waldman M, Jiang N, May HI, Hill TM, Luo Y, Yoo H, Zaha VG, Lavandero S, Gillette TG, Hill JA (2021) Xbp1s-FoxO1 axis governs lipid accumulation and contractile performance in heart failure with preserved ejection fraction. Nat Commun 12:1684. 10.1038/s41467-021-21931-9 33727534 10.1038/s41467-021-21931-9 PMC7966396

[46] Schiattarella GG, Altamirano F, Tong D, French KM, Villalobos E, Kim SY, Luo X, Jiang N, May HI, Wang ZV, Hill TM, Mammen PPA, Huang J, Lee DI, Hahn VS, Sharma K, Kass DA, Lavandero S, Gillette TG, Hill JA (2019) Nitrosative stress drives heart failure with preserved ejection fraction. Nature 568:351–356. 10.1038/s41586-019-1100-z 30971818 10.1038/s41586-019-1100-z PMC6635957

[47] Percie du Sert N, Hurst V, Ahluwalia A, Alam S, Avey MT, Baker M, Browne WJ, Clark A, Cuthill IC, Dirnagl U, Emerson M, Garner P, Holgate ST, Howells DW, Karp NA, Lazic SE, Lidster K, MacCallum CJ, Macleod M, Pearl EJ, Petersen OH, Rawle F, Reynolds P, Rooney K, Sena ES, Silberberg SD, Steckler T, Wurbel H (2020) The ARRIVE guidelines 2.0: updated guidelines for reporting animal research. PLoS Biol 18:e3000410. 10.1371/journal.pbio.3000410 32663219 10.1371/journal.pbio.3000410 PMC7360023

[48] Siri-Tarino PW, Sun Q, Hu FB, Krauss RM (2010) Meta-analysis of prospective cohort studies evaluating the association of saturated fat with cardiovascular disease. Am J Clin Nutr 91:535–546. 10.3945/ajcn.2009.27725 20071648 10.3945/ajcn.2009.27725 PMC2824152

[49] Stanley WC, Dabkowski ER, Ribeiro RF Jr., O’Connell KA (2012) Dietary fat and heart failure: moving from lipotoxicity to lipoprotection. Circ Res 110:764–776. 10.1161/CIRCRESAHA.111.253104 22383711 10.1161/CIRCRESAHA.111.253104 PMC3356700

[50] Taegtmeyer H, Young ME, Lopaschuk GD, Abel ED, Brunengraber H, Darley-Usmar V, Des Rosiers C, Gerszten R, Glatz JF, Griffin JL, Gropler RJ, Holzhuetter HG, Kizer JR, Lewandowski ED, Malloy CR, Neubauer S, Peterson LR, Portman MA, Recchia FA, Van Eyk JE, Wang TJ, American Heart Association Council on Basic Cardiovascular S (2016) Assessing cardiac metabolism: a scientific statement from the American Heart Association. Circ Res 118:1659–1701. 10.1161/RES.0000000000000097 27012580 10.1161/RES.0000000000000097 PMC5130157

[51] Tan Y, Li M, Wu G, Lou J, Feng M, Xu J, Zhou J, Zhang P, Yang H, Dong L, Li J, Zhang X, Gao F (2021) Short-term but not long-term high fat diet feeding protects against pressure overload-induced heart failure through activation of mitophagy. Life Sci 272:119242. 10.1016/j.lfs.2021.119242 33607155 10.1016/j.lfs.2021.119242

[52] Tavazzi L, Maggioni AP, Marchioli R, Barlera S, Franzosi MG, Latini R, Lucci D, Nicolosi GL, Porcu M, Tognoni G, Gissi HFI (2008) Effect of n-3 polyunsaturated fatty acids in patients with chronic heart failure (the GISSI-HF trial): a randomised, double-blind, placebo-controlled trial. Lancet 372:1223–1230. 10.1016/S0140-6736(08)61239-8 18757090 10.1016/S0140-6736(08)61239-8

[53] Toko H, Morita H, Katakura M, Hashimoto M, Ko T, Bujo S, Adachi Y, Ueda K, Murakami H, Ishizuka M, Guo J, Zhao C, Fujiwara T, Hara H, Takeda N, Takimoto E, Shido O, Harada M, Komuro I (2020) Omega-3 fatty acid prevents the development of heart failure by changing fatty acid composition in the heart. Sci Rep 10:15553. 10.1038/s41598-020-72686-0 32968201 10.1038/s41598-020-72686-0 PMC7512019

[54] Tuunanen H, Engblom E, Naum A, Nagren K, Hesse B, Airaksinen KE, Nuutila P, Iozzo P, Ukkonen H, Opie LH, Knuuti J (2006) Free fatty acid depletion acutely decreases cardiac work and efficiency in cardiomyopathic heart failure. Circulation 114:2130–2137. 10.1161/CIRCULATIONAHA.106.645184 17088453 10.1161/CIRCULATIONAHA.106.645184

[55] Wai T, Garcia-Prieto J, Baker MJ, Merkwirth C, Benit P, Rustin P, Ruperez FJ, Barbas C, Ibanez B, Langer T (2015) Imbalanced OPA1 processing and mitochondrial fragmentation cause heart failure in mice. Science 350:aad0116. 10.1126/science.aad0116 26785494 10.1126/science.aad0116

[56] Wende AR, Brahma MK, McGinnis GR, Young ME (2017) Metabolic origins of heart failure. JACC Basic Transl Sci 2:297–310. 10.1016/j.jacbts.2016.11.009 28944310 10.1016/j.jacbts.2016.11.009 PMC5609457

